# Splicing Factor PQBP1 Curtails BAX Expression to Promote Ovarian Cancer Progression

**DOI:** 10.1002/advs.202306229

**Published:** 2024-02-11

**Authors:** Xihan Liu, Jiaojiao Zhang, Zixiang Wang, Mingyao Yan, Meining Xu, Gaoyuan Li, Victoria Shender, Jian‐jun Wei, Jianqiao Li, Changshun Shao, Shiqian Zhang, Beihua Kong, Kun Song, Zhaojian Liu

**Affiliations:** ^1^ Key Laboratory of Experimental Teratology Ministry of Education Department of Obstetrics and Gynecology Qilu Hospital Department of Cell Biology School of Basic Medical Science Shandong University Jinan 250012 China; ^2^ Advanced Medical Research Institute Shandong University Jinan 250012 China; ^3^ Center for Precision Genome Editing and Genetic Technologies for Biomedicine Federal Research and Clinical Center of Physical‐Chemical Medicine of Federal Medical Biological Agency Moscow 119435 Russia; ^4^ Department of Pathology Northwestern University School of Medicine Chicago IL 60611 USA; ^5^ Department of Ophthalmology Qilu Hospital Shandong University Jinan 250012 China; ^6^ Key Laboratory of Radiation Medicine and Protection Institutes for Translational Medicine Soochow University Suzhou 215127 China

**Keywords:** alternative splicing, BAX, PQBP1

## Abstract

Splicing factor polyglutamine binding protein‐1 (PQBP1) is abundantly expressed in the central nervous system during development, and mutations in the gene cause intellectual disability. However, the roles of PQBP1 in cancer progression remain largely unknown. Here, it is shown that PQBP1 overexpression promotes tumor progression and indicates worse prognosis in ovarian cancer. Integrative analysis of spyCLIP‐seq and RNA‐seq data reveals that PQBP1 preferentially binds to exon regions and modulates exon skipping. Mechanistically, it is shown that PQBP1 regulates the splicing of genes related to the apoptotic signaling pathway, including BAX. PQBP1 promotes BAX exon 2 skipping to generate a truncated isoform that undergoes degradation by nonsense‐mediated mRNA decay, thus making cancer cells resistant to apoptosis. In contrast, PQBP1 depletion or splice‐switching antisense oligonucleotides promote exon 2 inclusion and thus increase BAX expression, leading to inhibition of tumor growth. Together, the results demonstrate an oncogenic role of PQBP1 in ovarian cancer and suggest that targeting the aberrant splicing mediated by PQBP1 has therapeutic potential in cancer treatment.

## Graphical abstract

Altered expression of splicing factors can lead to splicing reprogramming that contributes to tumor initiation and progression. Here, the authors report that PQBP1 promotes exon skipping and degradation of BAX, which inhibits apoptosis in ovarian cancer cells. Splice‐switching antisense oligonucleotides targeting BAX exhibit efficient antitumor activity, indicating a promising strategy for therapy of ovarian cancer.

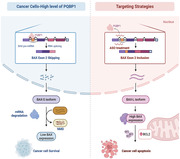

## 1 Introduction

RNA splicing is a key step during eukaryotic gene expression in which introns are removed and exons are ligated to generate mature mRNA.^[^ ^1^ ^]^ Alternative splicing (AS) enables a single multi‐exon gene to generate multiple isoforms, which increases transcriptome and proteome diversity.^[^ ^2^ ^]^ Approximately 95% of human multi‐exon genes undergo AS in order to produce variable isoforms in different cell and tissue types.^[^ ^3^ ^]^ AS plays important biological roles in development, differentiation, cell cycle progression, and cell death.^[^ ^4^ ^]^

AS is regulated by cis‐acting regulator elements and trans‐acting splicing factors.^[^ ^5^ ^]^ The 5′ splice site, 3′ splice site, and branchpoint sequence are canonical cis‐acting elements that define the exon/intron boundaries. Additional enhancer and silencer elements in exons and introns contribute to the specificity of AS regulation.^[^ ^6^ ^]^ Trans‐acting splicing factors play a pivotal role in the regulation of AS by binding to cis‐elements in pre‐mRNAs in order to activate or inhibit splicing.^[^ ^7^ ^]^ AS is a tightly controlled process, and its dysregulation has been implicated in several diseases, including cancer.

Recurrent somatic mutations in splicing factors, including SF3B1, U2AF1, and SRSF2, occur frequently in hematologic malignancies.^[^ ^8^ ^]^ In contrast, splicing factors exhibit frequent changes in copy number and expression level, but are rarely mutated in solid tumors.^[^ ^9^ ^]^ Emerging evidence indicates that splicing factors can act as proto‐oncogenes as well as tumor suppressors.^[^ ^10^ ^]^ For example, SRSF1 is frequently upregulated in various types of human cancer and its overexpression promotes the transformation of mammary cells,^[^ ^11^ ^]^ while SRSF6 promotes colorectal cancer progression through the aberrant splicing of ZO‐1.^[^ ^12^ ^]^ In addition to the SR proteins, NOVA1 promotes cell growth in non‐small cell lung cancer by regulating hTERT splicing,^[^ ^13^ ^]^ and MBNL3 plays an oncogenic role in hepatocellular carcinoma through the AS of lncRNA.^[^ ^14^ ^]^ Also, our previous studies showed that BUD31 promotes ovarian cancer progression by promoting exon inclusion in BCL2L12.^[^ ^15^ ^]^

PQBP1 is a nuclear polyglutamine‐binding protein that contains an N‐terminal WW domain.^[^ ^16^ ^]^ PQBP1 is abundantly expressed in the central nervous system during development,^[^ ^17^ ^]^ and a truncation mutation in PQBP1 causes intellectual disability.^[^ ^18^ ^]^ PQBP1 is an essential component of the cGAS /IRF3‐dependent innate response to HIV through the interaction with cGAS.^[^ ^19^ ^]^ In the nucleus, PQBP1 interacts with U5‐15 kD, a component of the U5 snRNP, which suggests a role for PQBP1 in splicing.^[^ ^20^ ^]^ A recent study has shown that high levels of PQBP1 are associated with poor prognosis in patients with osteosarcoma,^[^ ^21^ ^]^ which suggests its potential role in cancer progression, but the functional significance of PQBP1 in cancer remains largely unknown. Here, we show that ≈11% of ovarian cancer patients harbor genetic alteration of PQBP1 and that high levels of PQBP1 are associated with worse outcome. We further demonstrate that PQBP1 promotes ovarian cancer proliferation and xenograft tumor growth. Importantly, we show that PQBP1 regulates the AS and expression of BAX and that PQBP1 depletion leads to exon 2 inclusion that in turn upregulates BAX and triggers apoptosis in ovarian cancer cells. Our study thus suggests that PQBP1 acts as an oncogenic splicing factor that promotes the survival of ovarian cancer cells.

## 2 Results

### 2.1 PQBP1 is Frequently Increased and Indicates a Poor Prognosis in Ovarian Cancer

**Figure 1:**
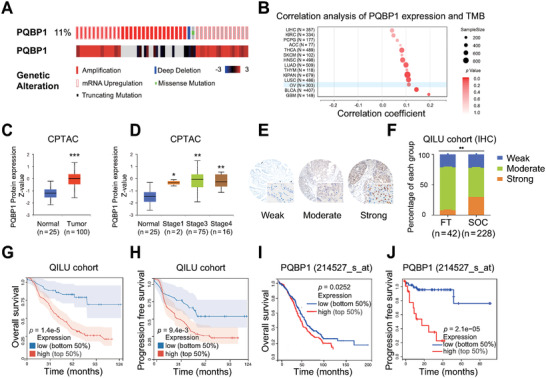
PQBP1 is frequently increased and indicates a poor prognosis in ovarian cancer. A) Genetic alteration of PQBP1 in ovarian cancer was obtained from cBioPortal data. Genetic alteration of PQBP1 (11% of all samples) includes 0.25% of missense mutation, 0.25% deep of deletion, 6.8% of gene copy number amplification, 6.0% of mRNA high expression, and 2.8% of both gene and mRNA amplification. B) The correlation between PQBP1 expression and tumor mutation burden was evaluated by Spearman's correlation analysis of the TCGA OV database (*n* = 303, Spearman coefficient R = 0.110, *P* < 0.05). C,D) PQBP1 protein expression level was analyzed in normal tissues and different stages of tumor tissues in the CPTAC database. E) Representative images of IHC staining of PQBP1 in tissue microarray from the Qilu Hospital cohorts (228 SOC tissues and 42 FT tissues). F) PQBP1 expression levels (weak, moderate, and strong) in FT tissues and SOC tissues in the tissue microarray. G,H) Kaplan‐Meier analysis of the correlation between PQBP1 protein level and patient overall survival (G)/progression‐free survival (H) in the Qilu Hospital cohort. I,J) Kaplan–Meier analysis of the correlation between PQBP1 mRNA expression and patient overall survival (I)/progression‐free survival (J) using the Kaplan Meier plotter cohort. The PQBP1 expression was divided into high and low groups using the best cutoff value. A two‐tailed test (B), Student's unpaired *t*‐test (C, D, and F), or Log‐rank test (G–J) were used to obtain the *P* value. ^*^ *P* < 0.05, ^**^ *P* < 0.01, ^***^ *P* < 0.001.

Our previous transcriptome (GSE135886) analysis show that 33 of 134 splicing factors were overexpressed in serous ovarian carcinoma (SOC) tissues compared with fallopian tube (FT) tissues.^[^ ^22^ ^]^ These splicing factors include PQBP1, which accounts for 11% of all genomic alterations (with 6.8% of gene copy number amplification and 6.0% of mRNA high expression) in SOC cases according to cBioPortal datasets (**Figure** **1**A). In addition, the increased PQBP1 expression was correlated with higher tumor mutational burden in TCGA OV cohort (Figure 1B). The UALCAN database ^[^ ^23^ ^]^ further showed higher protein levels of PQBP1 in cancer tissues, and its expression was related to clinical stage in ovarian cancer (Figure 1C,D), and pan‐cancer analysis showed that PQBP1 is overexpressed in various cancer types (Figure S1A, Supporting Information). We next performed immunohistochemistry (IHC) using a tissue microarray with 228 SOC tissues and 42 FT tissues and showed that PQBP1 was overexpressed in SOC tissues relative to FT tissues (Figure 1E,F). Importantly, the increased PQBP1 expression was correlated with advanced FIGO stage (P = 0.006), omental involvement (P = 0.002), and more deaths (P <0.001) (Table S2, Supporting Information). Multivariate Cox regression analysis further showed that high PQBP1 expression (P<0.001) and platinum resistance relapse (P = 0.017) were independent risk factors for reduced overall survival (Table S3, Supporting Information). Kaplan–Meier analysis showed that high PQBP1 expression in SOC tissue was correlated with worse overall survival and progression‐free survival both in our cohort and in the Kaplan‐Meier Plotter database (Figure 1G–J). Collectively, these results indicate that high PQBP1 expression is associated with poor prognosis in ovarian cancer patients.

### 2.2 PQBP1 Promotes the Proliferation and Xenograft Growth of Ovarian Cancer Cells

**Figure 2:**
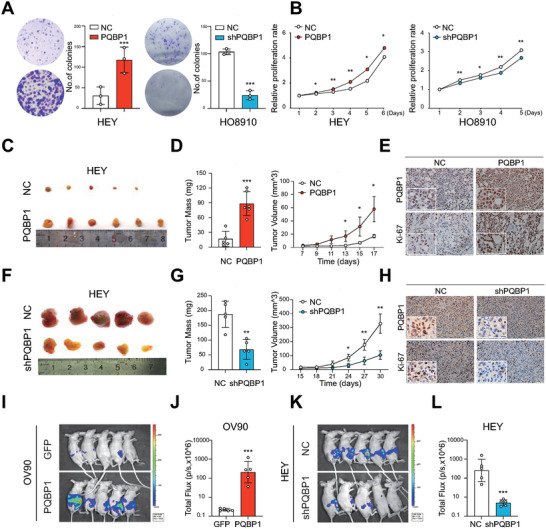
PQBP1 promotes the proliferation and xenograft growth of ovarian cancer cells. A,B) The effect of PQBP1 on ovarian cancer cell proliferation was analyzed via (A) colony formation assays and (B) MTT assays upon PQBP1 overexpression in HEY cells (*n* = 3) or knockdown in HO8910 cells (*n* = 3). C—E) Representative images of OV xenograft tumors (C), tumor mass and tumor volume (D), IHC staining of Ki‐67 and PQBP1 (E) in control and PQBP1 overexpressing HEY cells. F—H) Representative images of OV xenograft tumors (F), tumor mass and tumor volume (G), and IHC staining of Ki‐67 and PQBP1(H) in control and PQBP1 knockdown HEY cells. I,J) Images and quantification of luciferase signals in nude mice intraperitoneally injected with control and PQBP1‐overexpressing OV90 cells. K,L) Images and quantification of luciferase signals in nude mice intraperitoneally injected with control and PQBP1 knockdown HEY cells. NC group represents cells transfected with corresponding control vector. Student's unpaired *t*‐test was used to calculate the *p*‐value. Three independent experiments were performed to obtain the mean ± SD value (*n* = 3 replicate experiments). ^*^ *P* < 0.05, ^**^ *P* < 0.01, ^***^ *P* < 0.001.

To investigate the biological roles of PQBP1 in ovarian cancer, overexpression or knockdown of PQBP1 was established in several ovarian cancer cell lines (Figure S1B–E, Supporting Information). Colony formation and growth curve assays showed that ectopic expression of PQBP1 in HEY and A2780 cells significantly increased cell proliferation, while knockdown of PQBP1 exhibited the opposite effects (**Figure** **2**A,B; Figure S2A,B, Supporting Information). We further assessed the role of PQBP1 in ovarian cancer tumorigenesis in vivo. HEY cells with PQBP1 overexpression and control cells (n = 6) were injected subcutaneously into the flanks of nude mice, and tumor growth was measured over time. As shown in Figure 2C,D, overexpression of PQBP1 in HEY cells led to a significant increase in tumor mass and tumor volume. IHC staining showed that overexpression of PQBP1 increased the Ki‐67 proliferative index compared to the control group (Figure 2E). In contrast, knockdown of PQBP1 resulted in reduced tumor mass, tumor volume, and Ki‐67 proliferative index (Figure 2F–H). Moreover, luciferase‐expressing OV90 cells and HEY cells with PQBP1 overexpression or knockdown and corresponding control cells were intraperitoneally injected into nude mice (*n* = 5 for each group), and luciferase was used as a tracer for in vivo imaging analysis. Forced expression of PQBP1 significantly increased the number and size of abdominal tumors in the mice (Figure 2I,J), while knockdown of PQBP1 had the opposite effects (Figure 2K,L). These findings indicate that PQBP1 is involved in ovarian cancer progression.

### 2.3 Genome‐Wide Identification of PQBP1 Binding and PQBP1‐Regulated Splicing Events

**Figure 3:**
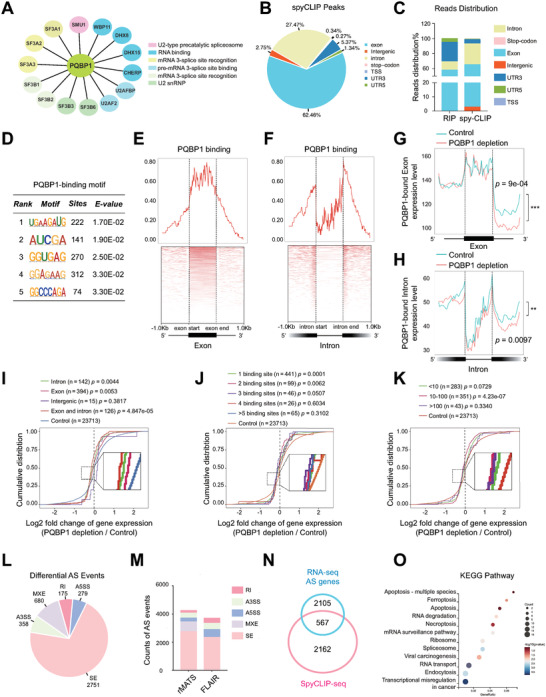
Genome‐wide identification of PQBP1‐regulated splicing events. A) PQBP1‐interacting spliceosome components (*n* = 15) were identified by immunoprecipitation coupled to mass spectrometry. B) Pie chart showing the distribution of PQBP1 binding peaks across different genomic regions using the spyCLIP‐seq data. C) Distribution of PQBP1 binding peaks in different genomic regions using RIP‐seq and the spyCLIP‐seq data. D) Top‐five PQBP1‐binding motifs were identified by STREME, and ranked by E‐value, and the binding site number is listed. The E‐value in STREME was calculated using a one‐sided binomial test. E—H) The PQBP1‐binding reads distribution (E,F) and transcripts (G, H) in genome‐wide regions by meta‐exon or meta‐intron analysis. The exon and intron region length were scaled to 1.0 kb to normalize different exon and intron lengths. Meta‐exon analysis of PQBP1 binding sites together with the heatmap of PQBP1 binding (E) and transcripts (G) in the PQBP1‐binding exons (*n* = 930). Meta‐intron analysis of PQBP1 binding sites together with the heatmap of PQBP1 binding (F) and transcripts (H) in the PQBP1‐bound introns (*n* = 409). *P*‐values were calculated by the Kolmogorov‐Smirnov test. I—K) The cumulative curves of gene expression changes were analyzed through combination analysis of the region (I), number (J), and enrichment (K) of PQBP1‐binding sites in HEY cells with PQBP1 depletion compared with control cells. P values were calculated by the pairwise Wilcoxon test with Bonferroni adjustment. L) Pie chart depicting the different types of significantly differential AS events upon PQBP1 depletion analyzed and filtered by rMATS. *P* < 0.05 and |IncLevelDifference| > 0.05 were the filter criteria. SE, skipped exon; RI, retained intron; A5SS, alternative 5′ splice site; A3SS, alternative 3′ splice site; MXE, mutually exclusive exons. M) Different AS events were analyzed by the rMATS using short‐read RNA‐seq data and algorithm FLAIR using long‐read Nanopore RNA‐seq data. N) Venn diagram of the differentially alternatively spliced genes analyzed by rMATS (2672) and the PQBP1‐bound genes (2729) acquired from the spyCLIP‐seq data. O) KEGG analysis of 567 overlapping genes from (N).

PQBP1 is a component of the precatalytic spliceosome (B complex) that is involved in splicing. ^[^ ^24^ ^]^ IHC and immunofluorescence analysis of SOC tissue samples and ovarian cancer cells showed that PQBP1 was predominantly localized in the cell nucleus (Figures 1E and 2E,H; Figure S3A, Supporting Information). Using immunoprecipitation coupled to mass spectrometry, we identified 15 core spliceosome components that interacted with PQBP1 in HEY cells (**Figure** **3**A; Table S4, Supporting Information), including the two well‐known PQBP1‐associated spliceosome components SF3B1 and WBP11.^[^ ^25^ ^]^ To gain global insights into PQBP1‐RNA binding sites, spyCLIP‐seq of PQBP1 was performed in HEY cells as described previously.^[^ ^26^ ^]^ The spy‐FLAG‐tagged version of PQBP1 was shown in Figure S3B (Supporting Information). By deep sequencing analysis of PQBP1‐bound RNAs, we found that PQBP1 was predominantly mapped to exons (62.46%) (Figure 3B). RIP‐seq analysis further verified that PQBP1 bound preferentially to exon regions (55.18%). (Figure 3C; Figure S3C, Supporting Information). The PQBP1‐binding motif is shown in Figure 3D. Next, we analyzed the PQBP1‐binding intensity across exons and introns and observed more binding intensity on exons (Figure 3E; Figure S3D, Supporting Information). Importantly, PQBP1 exhibited a strong binding preference for splicing sites, with greater binding to 5′ splicing sites (Figure 3F). The reads mapping distribution analysis showed that 3′ end introns adjacent to PQBP1‐bound exons were significantly decreased upon PQBP1 depletion (*p* = 0.0009, Figure 3G), while PQBP1‐bound introns demonstrated decreased intron retention and increased 3′ end exon skipping (*p* = 0.0097, Figure 3H). The genomic landscape of PQBP1‐binding on RNAs indicates its potential role in AS. Further, we compared PQBP1‐bound genes with differentially expressed genes upon PQBP1 knockdown and found that PQBP1‐bound genes were significantly downregulated on a genome‐wide scale (Figure 3I–K), suggesting that PQBP1 regulates gene expression by binding to exons.

We next analyzed the AS events using the rMATS software for short‐read RNA‐seq data in PQBP1 knockdown and control HEY cells. A total of 4243 differential AS events were identified, accounting for one‐third of the detected splicing events (Table S5, Supporting Information). Among differential AS events, exon skipping is the most prevalent type (64.84%, 2751 of 4243) (Figure 3L). A total of 1046 exon inclusion events and 993 exon skip events were identified upon PQBP1 depletion (Figure S3E, Supporting Information). Long‐read nanopore RNA sequencing data analysis using the FLAIR software further revealed that the most predominant AS events upon PQBP1 knockdown were skipped exons (63.41%, 2340 of 3690) (Figure 3M). To identify the direct targets that account for the function of PQBP1 in ovarian cancer, we compared PQBP1‐bound genes and differential AS events and identified 567 common genes (Table S6, Supporting Information). KEGG analysis of the overlapping genes showed pathways related to apoptotic signaling pathway. We subsequently measured the expression level of ten selected overlapping genes by qPCR in control and PQBP1 knockdown HEY cells. Consistent with the RNA‐seq data, the expression of genes including BAX, was verified by qPCR (Figure S3F, Supporting Information). Moreover, we analyzed the AS patterns and binding peaks of five differentially expressed potential targets that were bound with PQBP1 (Figure S4A—O, Supporting Information). We then successfully validated the AS and binding patterns of these genes by RT‐PCR, RIP‐seq and spyCLIP seq (Figure S4A–O, Supporting Information). These results imply that PQBP1 is a spliceosome component that regulates the splicing and expression of several genes.

### 2.4 Overexpression of PQBP1 Promotes Exon Skipping and Degradation of BAX

**Figure 4:**
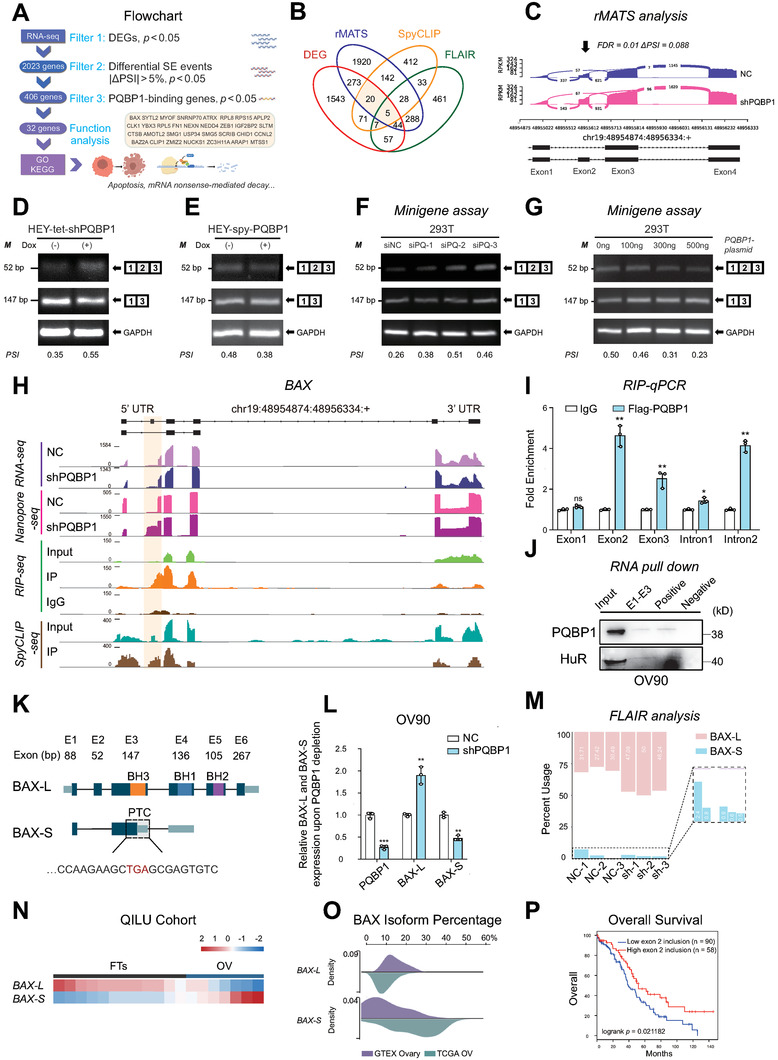
Overexpression of PQBP1 promotes exon skipping and degradation of BAX A) Flowchart summarizing the strategy of PQBP1 candidate target gene selection. B) The Venn diagram of differentially expressed genes from short‐read RNA‐seq data, differential AS events identified by rMATS and FLAIR and PQBP1 bound genes identified by spyCLIP‐seq data. C) IGV‐Sashimi plots showing the AS events in BAX using RNA‐seq data in HEY cells after PQBP1 depletion. The ΔPSI value for exon 2 skipping was calculated with MISO. D,E) RT‐PCR was performed to analyze BAX exon 2 inclusion upon PQBP1 knockdown (D) or overexpression (E) in HEY cells using isoform‐specific primers. F) A minigene assay was performed to verify BAX exon 2 skipping in 293T cells with PQBP1 depletion using different siRNAs. G) Minigene assay was conducted to verify BAX exon 2 skipping in 293T cells transfected with PQBP1 expression plasmids (0, 100, 300, 500 ng). H) AS of BAX and the PQBP1 bound region of BAX were visualized by IGV using RNA‐seq, Nanopore‐seq, RIP‐seq, and spyCLIP‐seq data. The orange region highlights the BAX AS and PQBP1 bounds region. I) RIP‐qPCR was performed to validate the binding regions of PQBP1 in BAX pre‐mRNA. J) RNA pull‐down assay verified the interaction between PQBP1 and BAX pre‐mRNA. The positive control was the 3′ UTR of the androgen receptor RNA. And the negative control was poly (A) _25_ RNA. K) Schematic diagram of two BAX isoforms. BAX‐L is the canonical isoform, and BAX‐S is the altered splicing isoform, which generates a premature termination codon (PTC). L) The relative mRNA expression of the two BAX isoforms was analyzed by qPCR in OV90 cells upon PQBP1 depletion. M) BAX‐L and BAX‐S percentages were analyzed by FLAIR using Nanopore‐seq data. N,O) BAX isoform percentages were analyzed in normal tissues and ovarian cancer tissues using Qilu Hospital cohort samples (N) and GTEx and TCGA databases (O). P) Analysis of relationship between BAX exon 2 inclusion level and overall survival using the best cutoff value. NC group represents cells transfected with corresponding control vector. Student's unpaired *t*‐test (I, L) or log‐rank test (P) was used to calculate the *p*‐value. Three independent experiments were performed to obtain the mean ± SD value (*n* = 3 replicate experiments). ^*^ *P* < 0.05, ^**^ *P* < 0.01, ^***^ *P* < 0.001.

We narrowed down PQBP1‐bound, alternatively spliced, and differentially expressed genes according to the flowchart shown in **Figure** **4**A. We took genes from the intersection of differentially expressed genes, differential AS events as identified by two algorithms (rMATS and FLAIR) and PQBP1‐bound genes as identified by spyCLIP (Figure 4B). Among the validated PQBP1‐bound and alternatively spliced genes, we focused on BAX, a pro‐apoptotic member of the BCL2 family. We analyzed the AS pattern of BAX in the RNA‐seq data using the rMATS algorithm and found that PQBP1 knockdown promoted exon 2 inclusion to generate the full‐length isoform of BAX (BAX‐L) without significant splicing events in other BAX genome locations (*p* < 0.05 and |IncLevelDifference| > 0.05) (Figure 4C; Table S5, Supporting Information). We next validated the AS pattern of BAX by RT‐PCR and found that PQBP1 depletion promoted exon 2 inclusion and generated a full‐length isoform of BAX (BAX‐L), while overexpression of PQBP1 promoted exon 2 skipping and generated a short isoform of BAX (BAX‐S) (Figure 4D,E). A minigene splicing assay further verified that siRNA knockdown of PQBP1 promoted exon 2 inclusion to generate BAX‐L (Figure 4F; Figure S5A, Supporting Information). In contrast, ectopic expression of PQBP1 promoted exon 2 skipping and resulted in BAX‐S (Figure 4G; Figure S5B, Supporting Information). To determine whether PQBP1 directly binds to BAX pre‐mRNA, we analyzed the spyCLIP‐seq and RIP‐seq data. Importantly, PQBP1 was shown to bind to exon 2 and intron 2 of BAX pre‐mRNA (Figure 4H). A RIP‐PCR assay further verified the direct binding of PQBP1 to BAX pre‐mRNA (Figure 4I). Moreover, an RNA pull‐down assay showed that PQBP1 was pulled down by the biotin‐labeled BAX pre‐mRNA (Figure 4J).

To clarify the consequence of PQBP1 on the AS of BAX, we analyzed the sequence of BAX‐S and found that exon 2 skipping introduced a premature termination codon that may result in nonsense‐mediated decay (Figure 4K). We next measured the RNA half‐life in HEY cells treated with Actinomycin D, and found that knockdown of UPF1 significantly prolonged the decay rate of BAX‐S, while BAX‐L was not affected (Figure S5C—E, Supporting Information). Overexpression of PQBP1 could abolish the accumulation of BAX caused by siUPF1 (Figure S5F, Supporting Information), while depletion of PQBP1 had the opposite effect (Figure S5G, Supporting Information). We further performed qPCR using isoform‐specific primers and found that knockdown of PQBP1 increased the level of BAX‐L, and decreased the level of BAX‐S, while overexpression of PQBP1 had the opposite effect (Figure 4L; Figure S5H, Supporting Information). Nanopore sequencing data analysis by FLAIR software further demonstrated that BAX‐L was increased and BAX‐S was decreased upon PQBP1 knockdown (Figure 4M); and BAX exon 2 skipping indeed was the major changed splicing event from PQBP1 knockdown (p < 0.05 and |IncLevelDifference| > 0.05 were used as thresholds) (Table S7, Supporting Information). Furthermore, we adopted kallisto program to analyze changes in all BAX isoforms from loss of PQBP1 function using Illumina and Nanopore data and arrived at the same conclusion that BAX‐L was increased and BAX‐S was decreased upon PQBP1 knockdown with other isoforms were not expressed or not significantly changed except two non‐protein coding transcripts BAX‐204 and BAX‐206 (Figure S5I, Supporting Information). Strikingly, BAX‐L was predominantly expressed in normal tissues, whereas BAX‐S was predominantly expressed in SOC tissues both in the Qilu Hospital and TCGA cohorts (Figure 4N,O). Moreover, ovarian cancer patients with low inclusion of exon 2 showed significantly poorer prognosis (Figure 4P). Taken together, these results indicate that PQBP1‐mediated AS and expression of BAX might play a role in ovarian cancer progression.

### 2.5 PQBP1 Depletion Upregulates BAX and Triggers Apoptosis in Ovarian Cancer Cells

**Figure 5:**
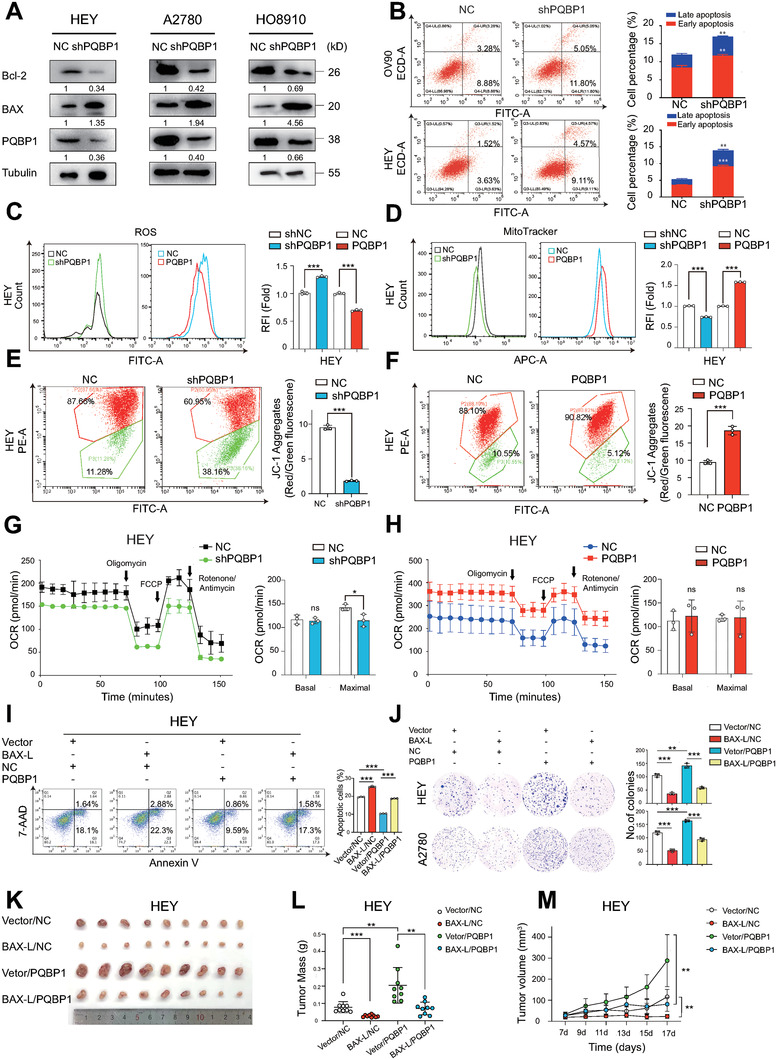
PQBP1 depletion upregulates BAX and triggers apoptosis in ovarian cancer cells. A) Immunoblotting was performed to measure Bcl‐2, BAX, and PQBP1 protein levels in three ovarian cancer cell lines upon PQBP1 depletion. B) Apoptotic cells were analyzed by flow cytometry using Annexin V/7‐AAD staining in OV90 and HEY cells with PQBP1 depletion. C) ROS were measured in ovarian cancer cells with PQBP1 knockdown or overexpression. D) Mitochondria mass was measured by flow cytometry after staining with Mito‐Tracker in ovarian cancer cells with PQBP1 knockdown or overexpression. E,F) Mitochondrial membrane potential was analyzed by flow cytometry followed by JC‐1 staining in ovarian cancer cells with PQBP1 depletion (E) or overexpression (F). G,H) OCR was measured under baseline conditions and after oligomycin, FCCP, and rotenone/antimycin injection, as indicated by the arrows. I—M) Apoptosis assays, colony formation assays, Xenograft experiments (*n* = 9) were conducted to assess the rescue effect of BAX on PQBP1 overexpression. NC group represents cells transfected with corresponding control vector. Vector group represents cells transfected with pcDNA3.1 plasmid. Student's unpaired *t*‐test was used to calculate the *p*‐value. Three independent experiments were performed to obtain mean ± SD value (*n* = 3 replicate experiments). ^*^ *P* < 0.05, ^**^ *P* < 0.01, ^***^ *P* < 0.001, ns: not significant.

To explore the functional significance of PQBP1‐mediated AS of BAX, we first performed immunoblotting and found that PQBP1 depletion significantly increased the level of BAX and decreased the level of BCL‐2 in OV cell lines (**Figure** **5**A). We then measured apoptosis using Annexin V‐PE/7‐AAD staining and flow cytometry and found that knockdown of PQBP1 expression was sufficient to induce spontaneous apoptosis of ovarian cancer cells (Figure 5B, Figure S6A, Supporting Information). In contrast, the overexpression of PQBP1 inhibited H_2_O_2_‐induced apoptosis of ovarian cancer cells (Figure S6B, Supporting Information). We measured the ROS levels and observed that PQBP1 knockdown significantly increased reactive oxygen species (ROS) levels while overexpression of PQBP1 suppressed ROS levels in HEY cells (Figure 5C). We further measured the mitochondrial mass using Mito‐Tracker staining and flow cytometry. The mitochondrial mass was significantly downregulated by PQBP1 knockdown and upregulated by PQBP1 ectopic expression (Figure 5D). Furthermore, the mitochondrial membrane potential was reduced in PQBP1 knockdown and was increased upon PQBP1 overexpression in HEY cells as evidenced by the JC‐1 assay (Figure 5E,F). Moreover, we measured respiratory capacity using Seahorse XF96 analyzer and found a significantly decreased maximal oxygen consumption rate (OCR) in PQBP1 knockdown cells (Figure 5G). However, there was no notable change in either basal or maximal OCR in PQBP1‐overexpressing cells (Figure 5H). These results indicate that PQBP1 depletion led to BAX upregulation and mitochondrial apoptosis in ovarian cancer cells.

To investigate the functional significance of PQBP1‐mediated AS of BAX, we performed rescue experiments by forced BAX‐L expression in PQBP1‐overexpressing and control HEY and A2780 cells (Figure S6C, Supporting Information). BAX‐L overexpression markedly reversed the inhibitor effects of PQBP1 on apoptosis (Figure 5I) and suppressed the PQBP1‐induced oncogenic potential as evidenced by colony formation and EdU assays (Figure 5J; Figure S6D, Supporting Information). Consistent with this, overexpression of BAX‐L significantly suppressed PQBP1‐induced xenograft tumor growth (Figure 5K–M). These results demonstrate that PQBP1 facilitates oncogenic potential partially by regulating the AS of BAX, to generate BAX‐S and thus inhibit apoptosis.

### 2.6 Splice‐Switching ASOs Targeting BAX Exhibit Efficient Antitumor Activity

**Figure 6:**
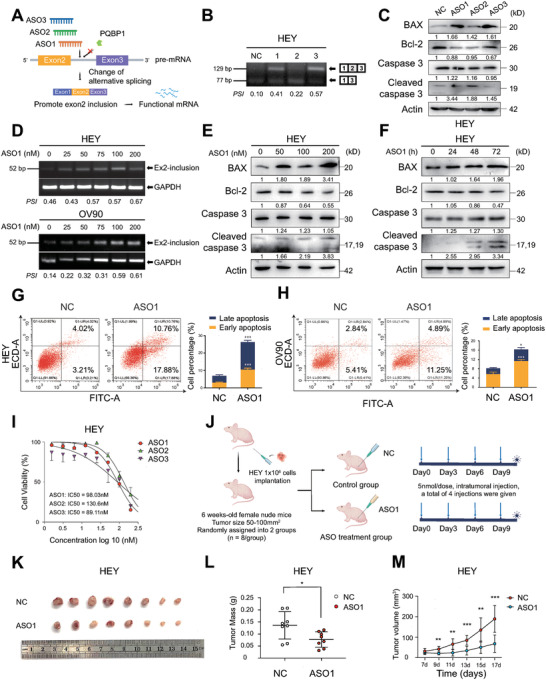
Splice‐switching ASOs targeting BAX exhibit efficient antitumor activity. A) The schematic diagram of designing ASOs targeting the AS of BAX. B) RT‐PCR was performed to analyze AS of BAX in HEY cells treated with three ASOs (200 nM) for 48 h. C) Immunoblotting was performed to measure the protein levels of BAX, Bcl‐2, and cleaved caspase 3 in HEY cells treated with three ASOs (200 nm) for 48 h. D) The inclusion of BAX exon 2 was analyzed by RT‐PCR in HEY and OV90 cells treated with different concentrations of ASO1 for 48 h. E) Immunoblotting was performed to measure the protein levels of BAX, Bcl‐2, and cleaved caspase 3 in HEY cells treated with ASO1 for gradient concentrations for 48 h. F) Immunoblotting was conducted to measure the protein levels of BAX, Bcl‐2, and cleaved caspase 3 in HEY cells treated with 200 nm ASO1 at 24, 48, and 72 h. G,H) Apoptotic cells were analyzed by flow cytometry after staining with Annexin V/7‐AAD in HEY (G) and OV90 cells (H) treated with ASO1 (200 nm) for 24 h. I) The IC_50_ of ASOs was calculated in HEY cells. J) The flow chart of ASO application in a tumor‐bearing nude mouse model. K—M) Images of xenograft tumors (*n* = 8 per group) (K), tumor weight (L), and tumor volume (M) in xenograft tumors of HEY cells after intratumoral injection of ASO1. NC group represents cells transfected with ASO‐control. Student's unpaired t‐test was used to calculate the *p*‐value. Three independent experiments were performed to obtain mean ± SD value (*n* = 3 replicate experiments). ^*^ *p* < 0.05, ^**^ *p* < 0.01, ^***^ *p* < 0.001.

We next sought to develop splice‐switching ASOs targeting BAX for the treatment of ovarian cancer. We designed ASOs to promote the inclusion of exon 2 of BAX based on the PQBP1 binding region in the pre‐mRNA of BAX (**Figure** **6**A). We synthesized three ASOs with phosphorothioate modifications and transfected them into HEY cells and analyzed BAX pre‐mRNA splicing by RT‐PCR. We found that ASO1 and ASO3 could induce splice‐switching of BAX, to generate a significant increase in BAX‐L (Figure 6B). We next performed immunoblotting and found that ASO1 treatment increased the BAX protein level in both HEY and OV90 cells (Figure 6C; Figure S7A, Supporting Information). ASO1 was the most potent ASO and was further evaluated in subsequent experiments. RT‐PCR further showed that ASO1 increased exon 2 inclusion in a concentration‐dependent manner (Figure 6D). Immunoblotting showed that 200 nM ASO1 significantly enhanced the protein level of BAX and cleaved Caspase 3 (Figure 6E) and increased apoptosis in a time‐dependent manner (Figure 6F; Figure S7B, Supporting Information). We further measured apoptosis using Annexin V‐PE/7‐AAD staining and found that 200 nM ASO1 triggered apoptosis in all three ovarian cancer cell lines tested (Figure 6G,H; Figure S7C–F, Supporting Information). Next, we calculated the half‐maximal inhibitory concentration (IC_50_) of ASO1 to be 98.03 nM in HEY cells (Figure 6I). Moreover, we evaluated the anti‐tumor effect of ASO1 in a xenograft model using HEY cells and found ASO1 treatment led to a significant reduction in tumor burden (Figure 6J–M). These results indicate that splice‐switching ASOs promote exon 2 inclusion, increase the protein level of BAX and inhibit ovarian tumor growth.

## 3 Discussion

PQBP1 has been studied extensively to be involved in neurodegenerative diseases^[^ ^25^ ^]^ and innate immunity. ^[^ ^19^ ^]^ A previous study has shown that high expression of PQBP1 is associated with metastasis and recurrence and predicts poor outcomes in osteosarcoma.^[^ ^21^ ^]^ However, the functional significance of PQBP1 in cancer has not been thoroughly studied. In the present work, we demonstrated that PQBP1 was frequently increased and indicated a poor prognosis in ovarian cancer. Pan‐cancer analysis using Clinical Proteomic Tumor Analysis Consortium (CPTAC) database revealed that PQBP1 is overexpressed in various cancer types including breast cancer, colon cancer, and glioblastoma. Subsequent investigations revealed that ectopic expression of PQBP1 promoted the development of ovarian cancer in vitro and in vivo. In contrast, PQBP1 depletion led to ROS accumulation, mitochondrial dysfunction, and finally induced apoptosis of ovarian cancer cells. Our findings provide strong evidence supporting the role of PQBP1 in ovarian cancer development.

In this work, we systematically investigated the global AS landscape regulated by PQBP1 through combined analysis of spyCLIP‐seq and RNA‐seq data, and found that PQBP1 was predominantly mapped to exons. Our study further demonstrated that the most predominant AS events upon PQBP1 knockdown were skipped exons. Previous studies have reported that PQBP1 regulates AS of BCL‐X in human A549 cells ^[^ ^27^ ^]^ and Ncam‐1 in mouse neurons,^[^ ^25^ ^]^ and our present work provides strong evidence that PQBP1 regulates AS of apoptotic genes, including BAX, in ovarian cancer cells. BCL2 family proteins frequently undergo AS to expand protein diversity.^[^ ^28^ ^]^ BAX consists of six exons and several isoforms have been previously reported, including canonical isoform BAX‐α and alternatively spliced isoforms ((Haferkamp et al.)). In contrast to BAX‐α, BAX‐β retains an un‐spliced intron 5, generating a distinct C‐terminal domain. BAX‐β forms a heterodimer with BAX‐α and promotes its change into an active conformation.^[^ ^29^ ^]^ BAX‐δ is a truncated isoform of BAX that results from the skipping of exon 3.^[^ ^30^ ^]^ BAX‐psi is an N‐terminal truncated isoform with partial deletion of exon 1, and the BAX‐psi protein is predominately localized to mitochondria and is a stronger inducer of apoptosis than BAX‐α.^[^ ^31^ ^]^ A recent study reported that SRSF6 deletion promotes the retention of BAX intron 1 to generate BAX‐k, an isoform that sensitizes macrophages to undergo apoptosis.^[^ ^32^ ^]^ In the present study, we demonstrated that PQBP1 knockdown promoted exon 2 inclusion to generate BAX‐L. In contrast, ectopic expression of PQBP1 promoted BAX exon 2 skipping and led to a premature termination codon and to a truncated isoform that underwent nonsense‐mediated decay.

Splice‐switching ASOs are promising approaches for anti‐cancer therapy. For example, ASOs that promote a shift from STAT3α to STAT3β exhibit good anti‐tumor activity both in vitro and in vivo,^[^ ^33^ ^]^ and ASOs that switch PKM splicing from the cancer‐associated PKM2 isoform to the PKM1 isoform reverse the Warburg effect and inhibit tumor growth.^[^ ^34^ ^]^ Also, our group previously developed ASOs that mediate BCL2L12 exon 3 skipping and suppress ovarian tumor growth.^[^ ^15^ ^]^ In this study, we developed ASOs based on the binding sites of PQBP1 in the pre‐mRNA of BAX. We demonstrated that the ASOs promoted exon 2 inclusion and generated BAX‐L, which in turn increased the protein level of BAX and induced apoptosis of ovarian cancer cells. Further study is needed to improve the specificity and delivery efficiency of these ASOs.

## 4 Conclusion

In conclusion, our work systematically studied PQBP1‐mediated AS and assessed the role of PQBP1 in ovarian cancer progression. We demonstrated that PQBP1 is commonly increased in SOC tissues and is correlated with poor prognosis. We also verified that PQBP1 promotes ovarian cancer progression by regulating the aberrant splicing and degradation of BAX. Of note, splice‐switching ASOs targeting BAX exhibited effective anti‐tumor activity. Thus, our study provides new insights into the oncogenic splicing factor PQBP1 in ovarian cancer development and suggests that PQBP1 represents a potential target for therapeutic interventions against ovarian cancer.

## 5 Experimental Section

### Patient Sample Collection

All evaluated FT and SOC tissues were obtained from the Department of Obstetrics and Gynecology, Qilu Hospital, Shandong University. SOCs (*n* = 228) used for tissue microarrays were collected from primary surgical operations without neoadjuvant therapy in 2007–2013. FTs (*n* = 42) were collected from patients with benign diseases through salpingectomy. Professional experts made the tissue microarrays in our lab. All patients provided informed consent before enrollment. The patient characteristics included the age, FIGO stage (2014), histology, Cancer Antigen 125 (CA‐125), tumor diameter, ascites involvement, omental involvement, the size of residual lesions which can be seen by naked eye after operation, adjuvant chemotherapy, platinum resistance relapse (relapse within 6 months after completion of platinum‐based therapy). Overall survival was the time interval from surgery to the death or last follow‐up. This study followed the principles of the Declaration of Helsinki and was approved by the ethics committee of Qilu Hospital, Shandong University (Date: 2021/12/20, KYLL‐202111‐073).

### Cell Lines and Cell Culture

A2780, HEY, SKOV3 cell lines were obtained from the Jian‐jun Wei lab, ^[^ ^35^ ^]^ Northwestern University. HO8910 cell line was purchased from the China Type Culture Collection, and OVCAR3, OV90, and HEK293T cell lines were purchased from the American Type Culture Collection. The SKOV3 cell line was grown in RPMI 1640 medium (Gibco, Thermo Fisher Scientific, USA) containing 10% fetal bovine serum (FBS) (Gibco) and 1% penicillin/streptomycin (Macgene), while the A2780, HO8910, HEK293T, HEY and OV90 cells were cultured in DMEM (Gibco, Invitrogen) containing 10% FBS and 1% penicillin/streptomycin. Standard cell culture conditions (37 °C, 5% CO_2_) in a humidified incubator were employed.

### Plasmid Construction and Cell Transfection

The doxycycline (Dox)‐inducible PQBP1 expression plasmid was constructed as described in previous study. ^[^ ^15^ ^]^ The sh‐PQBP1 sequence was cloned with a ClonExpress II One Step Cloning Kit (Vazyme) into the pGIPZ vector (Open Biosystem). The BAX minigene sequence was ligated into the pcDNA3.1 plasmid (Invitrogen). The lentiviral particles were harvested by co‐transfecting lentivirus plasmids with psPAX2 and pMD2.G into HEK293T cells. The lentivirus‐infected stable cell lines were screened out by puromycin (2 µg ml^−1^) selection for two weeks. PQBP1 and BAX small interfering RNA (siRNA) were synthesized by RiboBio. JetPRIME transfection reagent (PolyPlus) was used to transfect plasmids and siRNAs into cells. RNA and protein were extracted 48 or 72 h after siRNAs or plasmids transfection or Dox induction.

### RNA Isolation and PCR Analysis

A Cell Total RNA Isolation Kit (Foregene) was used to extract total RNA from cultured cells or fresh tissues, and the HiScript II Q RT SuperMix for qPCR Kit (Vazyme) was used to reverse transcribe total RNA into cDNA. The qPCR was performed with SYBR Green mix (Vazyme) on a QuantStudio 3 system (Applied Biosystems, USA). GAPDH was used as the endogenous control. The qPCR data were quantified by the 2^−ΔΔCt^ method. Semi‐quantitative RT‐PCR was used to analyze alternatively spliced isoforms. Primers were designed for the constitutive flanking exons, and 2 × Taq Master Mix (Dye Plus) (Vazyme, P112‐01) was used to amplify target exon spliced‐in or spliced‐out isoforms. The primer and ASO sequences used in this study are listed in Table S1 (Supporting Information).

### Western Blotting

Cells were placed on ice and lysed with lysis buffer (Beyotime, China), and the protein concentration was quantified with a BCA Assay Kit (Millipore, USA). Proteins were separated on 12% SDS‐PAGE gels and were then transferred to PVDF membranes (Millipore, USA) and blocked with 5% skim milk. The membranes were incubated in diluted primary antibodies at 4 °C overnight. Horseradish peroxidase (HRP)‐conjugated secondary antibodies and an ECL system (PerkinElmer, USA) were used to detect specific proteins the next day.

### Cell Proliferation and Colony Formation Assay

A methyl‐thiazolyl diphenyl‐tetrazolium bromide (MTT) assay was used to determine cell viability and proliferation. In brief, cells were seeded in 96‐well plates at a density of 1 × 10^3^ cells per well. At the observation time, cells were incubated with MTT (5 mg ml^−1^) at 37 °C for 4 h. Cell proliferation was observed over five days, and the IC_50_ of ASOs was measured 48 h after treatment. A total of 100 µl of dimethylsulfoxide (Sigma‐Aldrich, USA) was added to each well after discarding the supernatant, and the MTT‐formazan product was measured using a microplate reader (Bio‐Rad, Hercules, CA, USA) at 570 nm. A Cell‐Light EdU Apollo567 In Vitro Kit (RiboBio, Guangzhou, China) was used to perform the EdU cell proliferation assay according to the manufacturer's protocols. Cells were seeded at densities of 400 cells per well in six‐well plates and cultured for 2 weeks. crystal violet (0.1%, Solarbio) was used to stain colonies, and the number of colonies was counted by Image J.

### Nude Mouse Xenograft Model

Nude mouse xenograft experiment was performed as described previously. ^[^ ^15^ ^]^ Female BALB/c nude mice (6 weeks old) were randomly assigned to the indicated groups (*n* = 5‐8 mice of each group) and injected subcutaneously or intraperitoneally with PQBP1 overexpression or knockdown ovarian cancer cells. An imaging system (PerkinElmer) was used to capture bioluminescence images. ASO (Tsingke) treatment was performed by intratumor injection when the subcutaneous tumors in the mice reached 5 mm in diameter. All animal experiments were approved by the Shandong University Animal Ethics Research Board.

### IHC Staining

IHC staining of formalin‐fixed paraffin‐embedded xenograft tissue sections or tissue microarrays was performed with an IHC staining kit (ZSGB‐BIO). Paraffin sections were deparaffined with xylene and rehydrated with ethanol. EDTA buffer (pH 8.0) was used for antigen retrieval followed by goat serum blocking. The tissue sections were then incubated with primary antibodies anti‐PQBP1 (HPA001880, Sigma‐Aldrich) and anti‐Ki‐67 (#9449, CST) at 4 °C overnight. The sections were incubated with secondary antibody and stained with diaminobenzidine (DAB). The staining score of every sample was determined by two professional pathologists independently. A score of less than 7 represented low expression, and a score of 7 or greater represented high expression.

### Immunofluorescence

Cells were seeded on 24‐well glass coverslips and fixed with 4% paraformaldehyde on the observation day followed by permeabilization with 0.3% Triton‐X 100 (Solarbio, China). A 1% BSA solution was used for sample blocking at room temperature for 1 h. Primary antibodies against PQBP1 (HPA001880, Sigma‐Aldrich) and TOMM20 (11802‐1‐AP, Thermo Fisher Scientific) were incubated with the samples at 4 °C overnight. Samples were then washed three times with PBS containing 0.1% Triton X‐100 and incubated with secondary antibody for 1 h at room temperature. Nuclei were labeled with DAPI (Beyotime, China).

### Flow Cytometry Assay

An annexin V‐PE/7‐AAD Apoptosis Detection Kit (Vazyme, A213‐01) was used to detect cell apoptosis following the manufacturer's protocol. PQBP1 overexpressing cell lines were treated with H_2_O_2_ (400 µm) for 4 h before detecting cell apoptosis. Cells were analyzed within 20 min by CytoFLEX S (Beckman Coulter Life Science) after being incubated with Annexin V‐PE and 7‐AAD. A total of 1 × 10^4^ cells were used to calculate the percentage of apoptotic cells.

### ROS, Mitochondrial Mass, and Mitochondrial Membrane Potential Measurement

An oxidation‐sensitive fluorescent probe (DCFH‐DA) was used to detect ROS generation (Beyotime, China). PQBP1 knockdown or overexpression cells were induced by Dox for 48 h prior to 10 µm DCFH‐DA probe staining at 37 °C for 20 min. The cells were then washed three times with PBS and ROS were measured by flow cytometry (CytoFLEX S, Beckman Coulter Life Science). For mitochondrial mass measurement, cells were incubated with 100 nm Mitotracker Red for 30 min at 37 °C and, then washed three times with preheated medium and measured by flow cytometry. A Mitochondria Staining Kit (JC‐1, MultiSciences Biotech, China) was used to determine the mitochondrial membrane potential. Cells were incubated with JC‐1 (2 µm) for 20 min at 37 °C followed by flow cytometry. A total of 1 × 10^4^ viable cells were analyzed.

### Seahorse Mitochondrial Stress Assay

The OCR was determined using a Seahorse XF cell mitochondrial stress test kit (Seahorse Bioscience, USA). Control and PQBP1 knockdown or overexpression ovarian cancer cells were seeded on XFe96 plates (Agilent Technologies, Santa Clara, USA) at a density of 1 × 10^4^ cells per well and grown overnight in DMEM with 10% serum at 37 °C and, 5% CO_2_. On the experimental day, the cell culture medium was changed to XF Assay Medium (Agilent Technologies, Santa Clara, USA) at pH 7.4, supplemented with 10 mm glucose, 1 mm sodium pyruvate, and 2 mm glutamine, and incubated for 1 h at 37 °C in the absence of CO2. Cellular OCR measurements were recorded at baseline and then the cells were challenged with injections of the following inhibitors into the Seahorse ports: 1.5 m oligomycin, 2 m FCCP, 0.5 m rotenone, 0.5 m antimycin, 4 m Etomoxir, 2 m UK5009, and 3 m BPTES. Using Hoechst staining, the data from the Seahorse assays were standardized with cell counts (B8040, Solarbio). The Seahorse Wave program was used to analyze the data (Agilent Technologies, Santa Clara, USA). Each cell line underwent a minimum of two Seahorse replications, and each cell line had a minimum of six wells in each replicate.

### Immunoprecipitation Mass Spectrometry

PQBP1‐interactome was detected by immunoprecipitation coupled to mass spectrometry as described in our previous study. ^[^ ^22^ ^]^ FLAG‐tagged PQBP1 overexpressing HEY cells were lysed on ice using Western and IP Cell Lysis Buffer (WIP buffer, Beyotime Institute of Biotechnology), and 5 µg FLAG antibody (CST) was added to whole‐cell extracts for incubation for 1 h, followed by incubation with magnetic Protein A/G beads (Bimake) overnight at 4 °C. Beads were washed three times with WIP buffer, and immunocomplex were separated on SDS‐PAGE Coomassie‐stained gel. Protein bands were excised from the gel, and tandem liquid chromatography/mass spectrometry was used to detect the peptides.

### Short‐Reads RNA‐seq

Total RNA was extracted from cells with TRIzol reagent (Invitrogen) and total RNA was quantified and analyzed by Agilent Bioanalyzer 2100 and RNA 1000 Nano LabChip Kit (Agilent, CA, USA) with an RNA integrity number >7.0. Poly‐T oligo‐attached magnetic beads were used to purify poly(A) mRNA by two rounds of purification. The mRNA was fragmented and reverse‐transcribed to construct the cDNA library using the mRNASeq sample preparation kit protocol (Illumina, San Diego, USA); and the cDNA library was paired‐end sequenced on an IlluminaHiseq4000 following the protocol. Clean reads were mapped to the hg38 human genome by HISAT2 (version 2.2.0) and sorted by samtools (version 1.9). Mapped reads were visualized with the Integrative Genomics Viewer (IGV). StringTie (1.3.0) was used to reconstruct transcripts, and edgeR was used to analyze differential gene expression. Genes with |Fold change| > 2 and p <0.05 were considered to be differentially expressed. The short‐reads RNA‐seq data were deposited in the Gene Expression Omnibus (GEO) under accession code GSE223091.

### Nanopore Long‐Reads RNA‐seq

A total of 1 µg total RNA was used for cDNA library construction according to the Oxford Nanopore Technology (ONT) protocol. Full‐length mRNAs were reverse transcribed using the SuperScript IV First‐Strand Synthesis System (Invitrogen) and amplified for 14 cycles with LongAmp Tag (NEB). The PCR products underwent the formalin‐fixed paraffin‐embedded DNA repair and end‐repair (NEB) steps followed by adaptor ligation using T4 DNA ligase (NEB). DNA purification was performed using Agencourt XP beads according to ONT protocol. The cDNA libraries were run on a PromethlON platform at the Biomarker Technology Company (Beijing, China). Thresholds for raw reads filtration had a minimum average read quality score of 7 and a minimum read length of 500 bp, and ribosomal RNA was discarded. Full‐length, non‐chimeric transcripts were obtained after mapping to the hg38 genome using minimap2 (version 2.18) and removing redundant transcripts with the cDNA Cupcake package. After polishing with pinfish, consensus isoforms were obtained. Differentially expressed genes were obtained with the DESeq R package (1.18.0), and significantly different expression was filtered out by |Fold change| > 2 and p <0.05. The Nanopore long‐reads RNA‐seq data were deposited in the Gene Expression Omnibus under accession code GSE223088.

### Identification of AS Events

rMATS (version 4.1.0) was used to calculate the splicing variants from the RNA‐seq data. Thresholds for significant AS events were *p* < 0.05 and |IncLevelDifference| > 0.05. A sashimi plot was used to draw the diagrams. FLAIR (version 1.5) was used to identify AS events and calculate the splicing variants in the Nanopore‐seq data with the following parameters: diffSplice‐drim1 3‐drim2 3 test. DRIMSeq was used to perform statistical tests between groups to identify differential AS events (*p* < 0.05). A log2 fold change of gene expression after the Cufflinks status test was used for cumulative plots. Cumulative distribution was calculated with the ddply package. The pairwise Wilcoxon test with Bonferroni adjustment was used to calculate p values in the cumulative plots, and the data were visualized using the ggplot2 package in R.

### RIP‐seq

FLAG/Spy‐tagged PQBP1 overexpressing HEY cells were used for RIP‐seq by using the EZ‐Nuclear RIP (Cross‐Linked) Kit (Merck Millipore). Briefly, cells were harvested and cross‐linked by 0.3% formaldehyde. Nuclei were extracted and lysed in nuclear lysis buffer, and DNA was subjected to sonication to shear it into 500–1000 bp fragments. Magnetic beads with anti‐FLAG antibody (Cell Signaling Technology) were incubated with the nuclear lysates, and DNaseI was used to degrade the DNA. The library was constructed and pair‐end sequenced by Novogene Corporation (Beijing, China) on an Illumina platform (Illumina, CA, USA). An agilent Bioanalyzer 2100 system was used to assess library quality. Fastp software (version 0.19.11) was used to process raw data into clean data. Clean reads were aligned to the hg38 genome by BWA mem (v 0.7.17), and MACS2 peak calling software (version 2.1.0) was used to identify immunoprecipitation enrichment of the genomic regions (q‐value < 0.05). Peak‐ related genes were annotated by PeakAnnotator, and HOMER was used to detect the denovo motif sequences. The RIP‐seq data were deposited in the Gene Expression Omnibus under accession code GSE223090.

### SpyCLIP‐seq

Genome‐wide PQBP1‐RNA interactions were identified by SpyCLIP‐seq as described previously. ^[^ ^26^ ^]^ FLAG/Spy‐tagged PQBP1 overexpressing HEY cells were irradiated at 400 mJ cm^−2^ using a UV Crosslinker (UVP, CL‐1000). A Nucleoprotein Extraction Kit (Sangon) was used to extract nuclei prior to lysis. DNA was removed using Turbo DNase (2 U/µl, Invitrogen, AM2238) and RNA was fragmented with 1:200 diluted RNase I (100 U µl^−1^, Invitrogen, AM2295). Anti‐FLAG magnetic beads (MBL, M185‐11) were added to the mixed lysate for 1 h at 25 °C, and the beads were washed stringently and digested with proteinase K (Roche, 3115828001). RNA was purified using a Spin Column RNA Cleanup & Concentration Kit (Sangon), and the library was constructed and sequenced by the Ribobio Biotechnology Company using the NEB Next Ultra RNA Library Prep Kit for Illumina and the PE150 strategy (Novogene) on a HiSeq 2500. Trimmomatictools (version 0.36) was used to trim the adaptors and low‐quality bases, and RNAcentral (rnacentral.org/) was used to obtain clean reads by deleting ribosomal RNA. Then clean reads were mapped to the hg38 genome with the TopHat software (v2.0.13), and IGV was used to visualize the mapped reads. Piranha software (version 1.2.1) was employed to perform peak calling, and HOMER (version 4.11) was used to annotate the peaks. STREME (version 5.2.0) and MEME (version 5.3.0) were used to detect the consensus motif, and motif central enrichment was performed by CentriMo (version 5.3.0).

For calculation of meta‐exons or meta‐introns, each PQBP1‐binding exon or intron with a length of more than 100 bp was divided into 100 bins by bedtools. The format of the RNA‐seq data was converted to bigwig by wigToBigWig. After the reads were counted in each bin using bigWigAverageOverBed, the average counts in each bin were calculated by adjusting the direction of the genes. The heatmap was rendered by R with the pheatmap package. P values were calculated by the Kolmogorov–Smirnov test. Correlation of the spyCLIP data was calculated using genome‐wide 10000‐bp windows. The data were visualized in R with the ggplot2 package. The spyCLIP‐seq data were deposited in the Gene Expression Omnibus under accession code GSE223196.

### Minigene Constructs

The BAX exon 1 to exon 4 fragment was cloned into pcDNA 3.1. Plasmids containing BAX exon 1 to exon 4 and PQBP1 or negative control small interfering RNAs (siRNAs) were co‐transfected into HEK293T and HeLa cells. After 48 h, RNA was extracted and reverse‐transcribed into cDNA. Semi‐quantitative RT‐PCR was used to analyze alternatively spliced isoforms as previously described, and the PCR products were examined by DNA agarose gel electrophoresis.

### RNA Pull‐Down Assay

BAX pre‐mRNA fragments were ligated to T7 promoters, and an RNAMAX‐T7 In Vitro Transcription Kit (RiboBio) was used to transcribe the biotin‐labeled BAX RNA fragments using the Pierce RNA 3′ End Desthiobiotinylation Kit (Thermo Fisher Scientific). A magnetic RNA Protein Pull‐Down Kit (Thermo Fisher Scientific) was used to perform RNA pull‐down. The negative control was poly (A) _25_ RNA, and the positive control was the 3′ UTR of the androgen receptor RNA.

### Bioinformatics

The CPTAC databases were used to obtain pan‐cancer protein expression profiles, and cBioPortal (https://www.cbioportal.org/) was used to assess the correlations between PQBP1 expression levels with clinical parameter features in ovary carcinoma datasets (TCGA, provisional). Tumor mutation burden in tumors was analyzed by maftools (version 2.8.05), and patients were stratified as low and high PQBP1 expression groups using the median or best cutoff value. The patient overall survival and progression‐free survival were determined by Kaplan‐Meier survival analysis. Isoform percentages were viewed on UCSC Xena, and PSI values of BAX exon 2 in ovarian cancer were obtained from TCGASpliceseq. Gene Ontology (GO) enrichment analysis and Kyoto Encyclopedia of Genes and Genomes (KEGG) analysis were performed using the GOseq R packages and KOBAS software.

### Statistical Analysis

SPSS Statistics (version 26.0) and GraphPad Prism (version 9.0) were used to perform statistical analysis. In the analysis of tissue samples and clinical information, continuous variables such as patient age, CA‐125, and tumor size were converted to binary data for further analysis using median cutoff. The chi‐square test, student's *t*‐test, and one‐way ANOVA were used to determine significance. Univariate and multivariate Cox proportional hazard regression analysis was used to analyze high‐risk factors related to OS. R software packages (version 4.1.0) were used to analyze the differences in clinical values. Results are shown as the mean ± SD of three independent experiments. The significance threshold was set at *p* < 0.05.

## Conflict of Interest

The authors declare no conflict of interest.

## Supporting information

Supporting Information

Supplemental Table 1

Supplemental Table 2

Supplemental Table 3

Supplemental Table 4

Supplemental Table 5

Supplemental Table 6

Supplemental Table 7

## Data Availability

The data that support the findings of this study are available from the corresponding author upon reasonable request.
