# Supplementary material for: Splicing Factor PQBP1 Curtails BAX Expression to Promote Ovarian Cancer Progression
Source: Adv Sci (Weinh). 2024 Feb 11;11(15):2306229. doi: 10.1002/advs.202306229 (PMC11022708; doi:10.1002/advs.202306229)
Supplement: Supplementary file 1 — Supporting Information [file ADVS-11-2306229-s002.pdf]

ADVANCED SCIENCE Open Access

Supporting Information

for *Adv. Sci.*, DOI 10.1002/advs.202306229

Splicing Factor PQBP1 Curtails BAX Expression to Promote Ovarian Cancer Progression

Xihan Liu, Jiaojiao Zhang, Zixiang Wang, Mingyao Yan, Meining Xu, Gaoyuan Li, Victoria Shender, Jian-jun Wei, Jianqiao Li, Changshun Shao, Shiqian Zhang, Beihua Kong, Kun Song and Zhaojian Liu**

Splicing factor PQBP1 curtails BAX expression to promote ovarian cancer progression

Xihan Liu[1, 2], Jiaojiao Zhang[1], Zixiang Wang[1, 2], Mingyao Yan[1], Meining Xu[1], Gaoyuan Li[1], Victoria Shender[3], Jian-jun Wei[4], Jianqiao Li[5], Changshun Shao[6], Shiqian Zhang[1], Beihua Kong[1], Kun Song[1], Zhaojian Liu[1,2*]*

Figure S1, related to Fig. 1-2

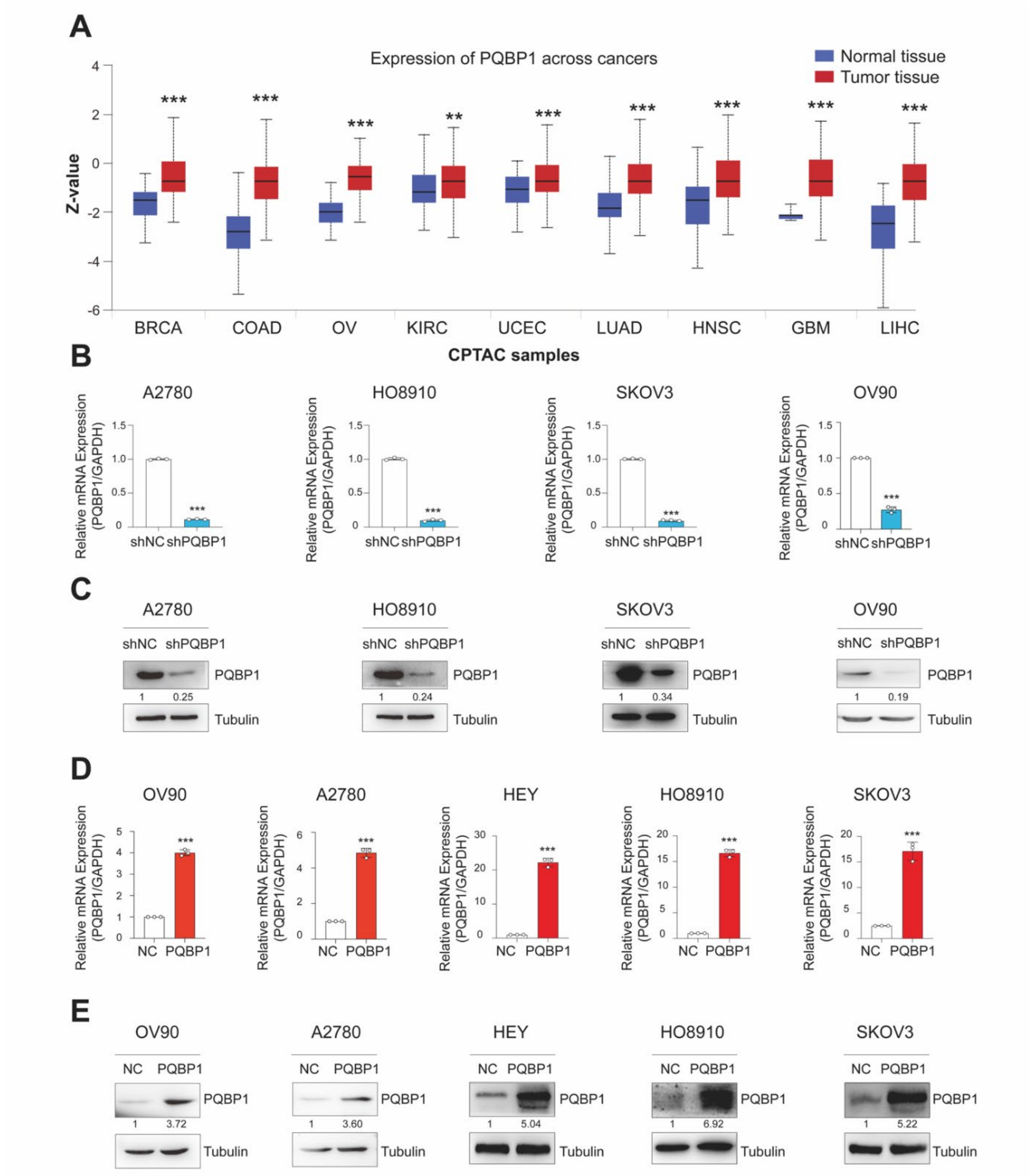

Fig. S1 The expression of PQBP1 in the TCGA tumors and construction of cell lines, related to Fig. 1-3. A PQBP1 protein expression level across cancers between normal tissues and tumors by CPTAC database. Z-score represent standard deviations from the median across samples for the given cancer types. **B** PQBP1 mRNA level in stably PQBP1-depleted OV cell lines. **C** PQBP1 protein level in stably PQBP1-depleted OV cell lines. **D** PQBP1 mRNA level in stably PQBP1-overexpressed OV cell lines. **E** PQBP1 protein level in stably PQBP1-overexpressed OV cell lines. Student's unpaired t- test was used to calculate P value. Three independent experiments were performed to gain mean ± SD value (n = 3 replicate experiments). *$P < 0.05$, **$P < 0.01$, ***$P < 0.001$.

Figure S2, related to Fig. 2

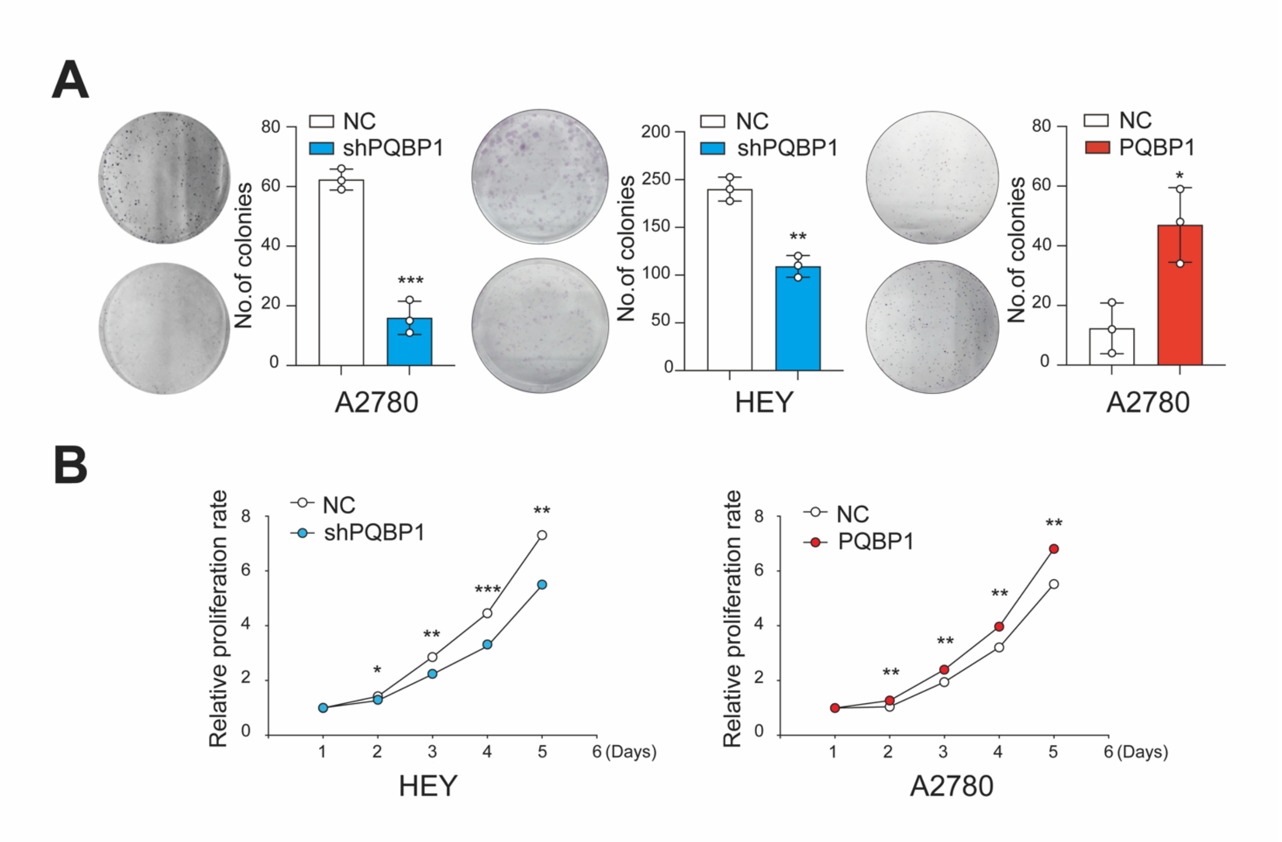

Fig. S2 PQBP1 promotes proliferation of ovarian cancer cells, related to Fig. 3. A-B Colony formation assay **(A)** and MTT assay **(B)** on PQBP1-depleted or overexpressed OV cell lines. P value was calculated by student's unpaired t-test.

Three independent experiments were performed to gain mean ± SD value (n = 3 replicate experiments). **P* < 0.05, ***P* < 0.01, ****P* < 0.001.

Figure S3, related to Fig. 3

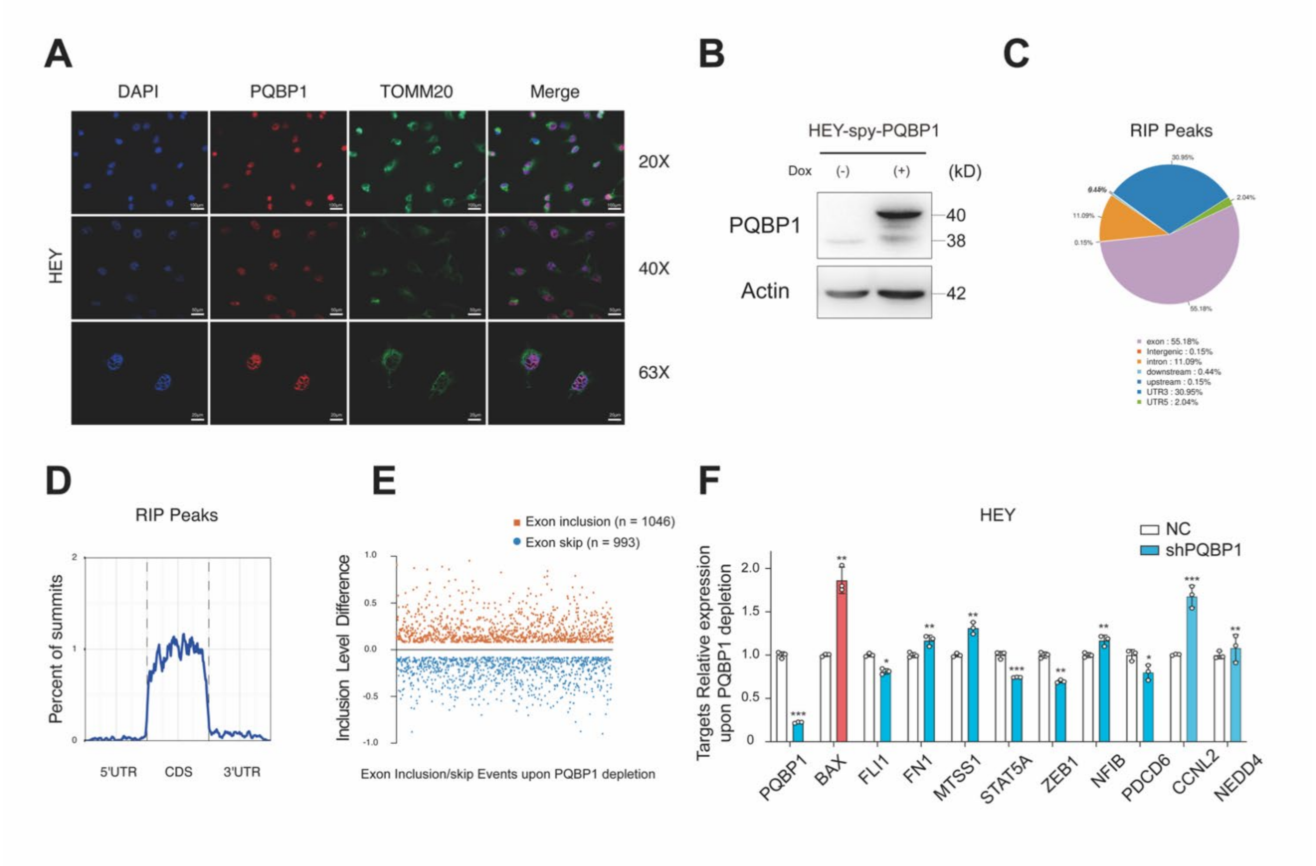

Fig. S3 Genome-wide identification of PQBP1 regulated splicing events and gene expression, related to Fig. 4. **A** Immunofluorescence experiments showed the nuclear localization of PQBP1 (red) and the mitochondrial localization indicated by mitochondrial marker TOMM20 (green). **B** The overexpression efficiency of spy-FLAG-tagged PQBP1 in HEY-spy-PQBP1 cells by Western blot. **C** Pie chart showing the proportions of PQBP1-binding sites identified by RIP on genome elements. **D** RIP read distribution on the genome elements, including 5' UTR, CDS region and 3' UTR. **E** The number of exon inclusion and skip events in stable PQBP1-depleted HEY cells induced by Dox treatment for 48 h. **F** The relative mRNA expression of PQBP1-target genes compared with corresponding control by qPCR analysis upon PQBP1 depletion in OV90 cells. P value was obtained by student's unpaired t-test. Three independent experiments were performed to gain mean ± SD value (n = 3 replicate experiments). *$P < 0.05$, **$P < 0.01$, ***$P < 0.001$.

Figure S4, related to Fig. 3

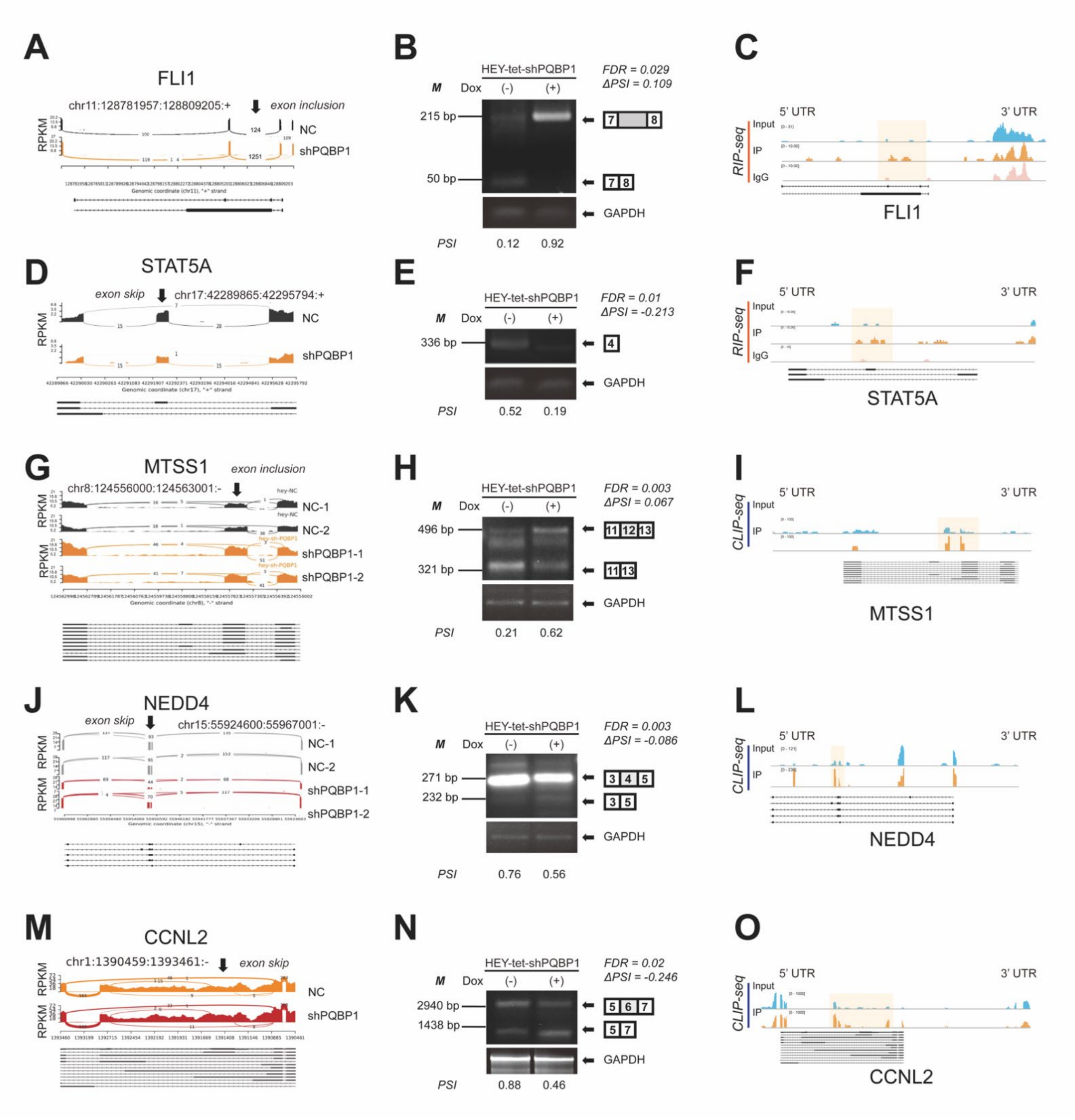

Fig. S4 The visualization of target gene splicing patterns and binding sites regulated by PQBP1, related to Fig. 4. **A-O** AS pattern of DEGs were verified by rMATs software based on RNA-seq data **(A, D, G, J, M)**, fragment analysis with semi-quantitive RT-PCR **(B, E, H, K, N)**. Genes with PQBP1 direct binding sites identified by RIP and spyCLIP were visualized with IGV **(C, F, I, L, O)**. The orange region highlights the PQBP1 binding region.

Figure S5, related to Fig. 4

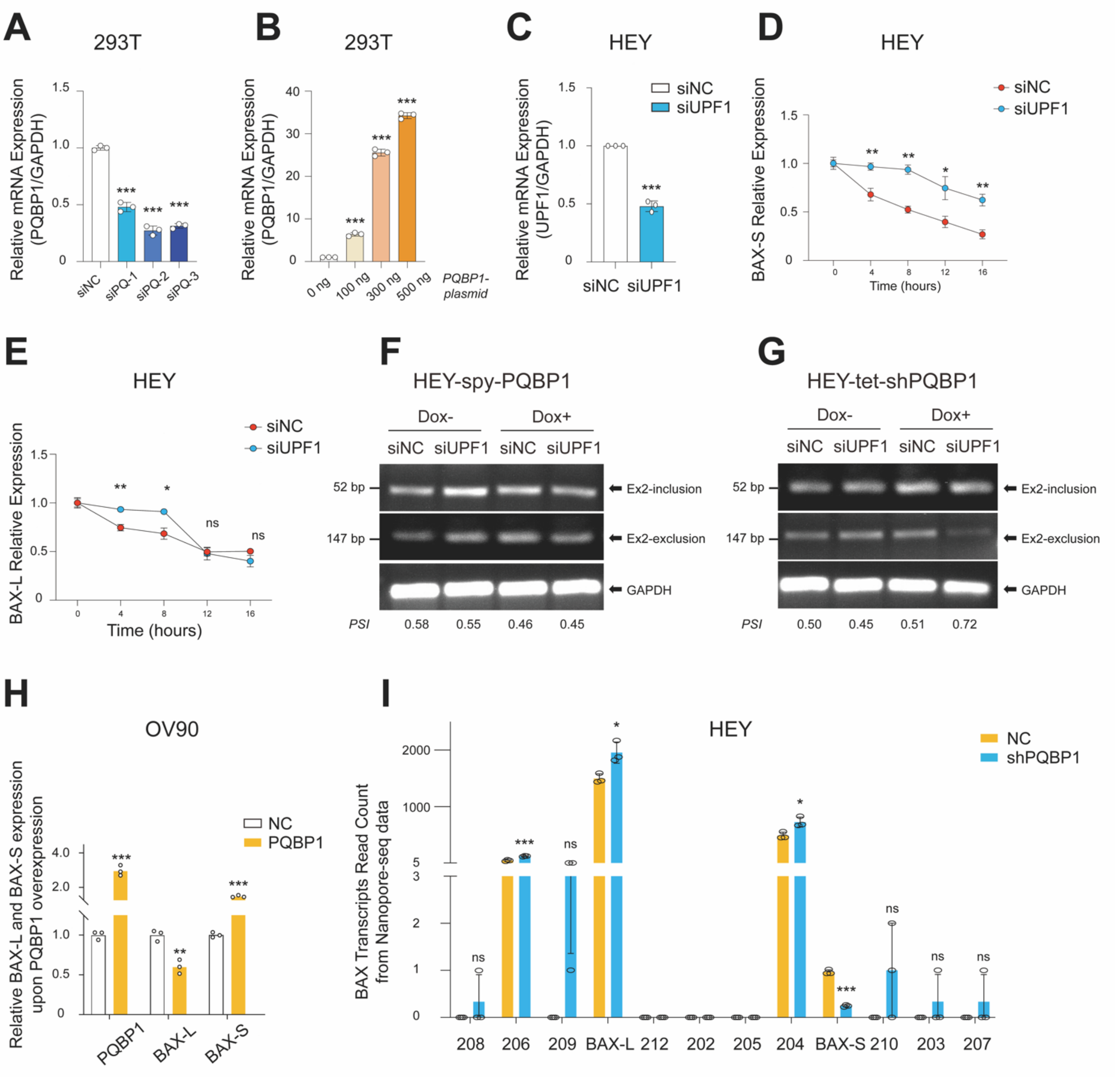

Fig. S5 The alternative splicing pattern of BAX regulated by PQBP1, related to Fig. 5. A-B PQBP1 knockdown efficiency **(A)** and PQBP1 overexpression efficiency **(B)** in 293T cells. **C-E** UPF1 knockdown efficiency **(C)** and remaining BAX-S **(D)** and BAX-L **(E)** mRNA expression in UPF1 depletion or control HEY cells at 0h, 4h, 8h, 12h and 16h after 10μg/ml actinomycin D treatment were analyzed by qPCR. **F-G** Semi-quantitive RT-PCR analysis of the BAX transcript variants following NMD pathway inhibition via siRNA-mediated depletion of the regulator UPF1 with or without PQBP1 overexpression (OE) **(F)** or depletion **(G)** in HEY cells. **H** The relative mRNA expression of two BAX transcript variants compared with corresponding control by qPCR analysis upon PQBP1 overexpression in OV90 cells. **I** The read count of all BAX transcript variants from Nanopore-seq data upon PQBP1 knockdown in HEY cells. P value was obtained by student's unpaired t-test. Three independent experiments were performed to gain mean ± SD value (n = 3 replicate experiments). *P < 0.05, **P < 0.01, ***P < 0.001.

Figure S6, related to Fig. 5

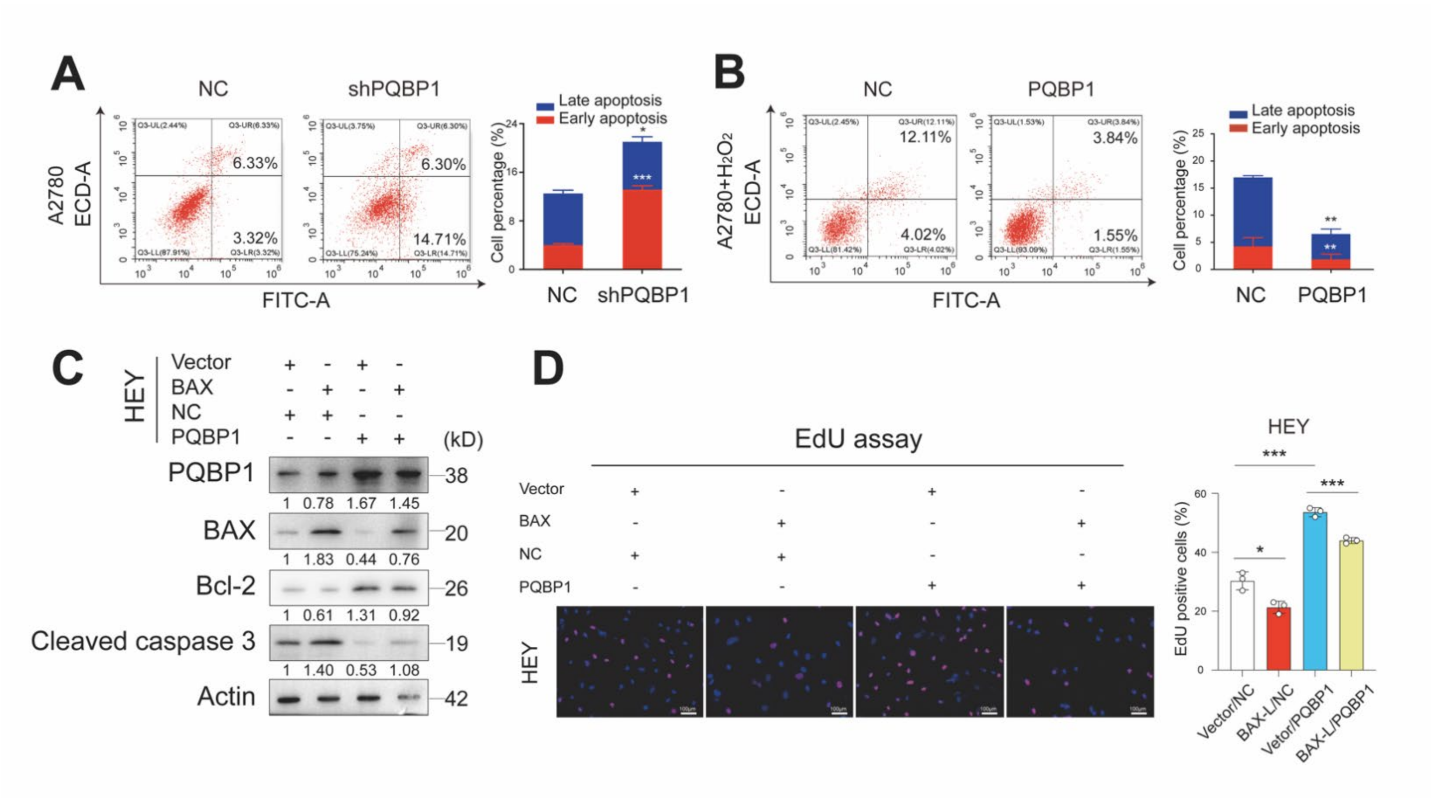

Fig. S6 PQBP1 depletion upregulates BAX and triggers apoptosis of ovarian cancer cells, related to Fig. 6. A-B The cell apoptosis in OV cells with PQBP1 depletion **(A)** and overexpression **(B)** was judged by Annexin V/7-AAD Apoptosis Detection assay followed by flow cytometry. **C** Western blot analysis of BAX and related apoptosis biomarkers, as well as PQBP1 protein expression in HEY cells with or without BAX or PQBP1 overexpression vector. **D** Overexpression of PQBP1 partially rescued the effects of BAX overexpression on cell proliferation in HEY cells. P value was calculated by student's unpaired t-test. Three independent experiments were performed to gain mean ± SD value (n = 3 replicate experiments). **P* < 0.05, ***P* < 0.01, ****P* < 0.001.

Figure S7, related to Fig. 6

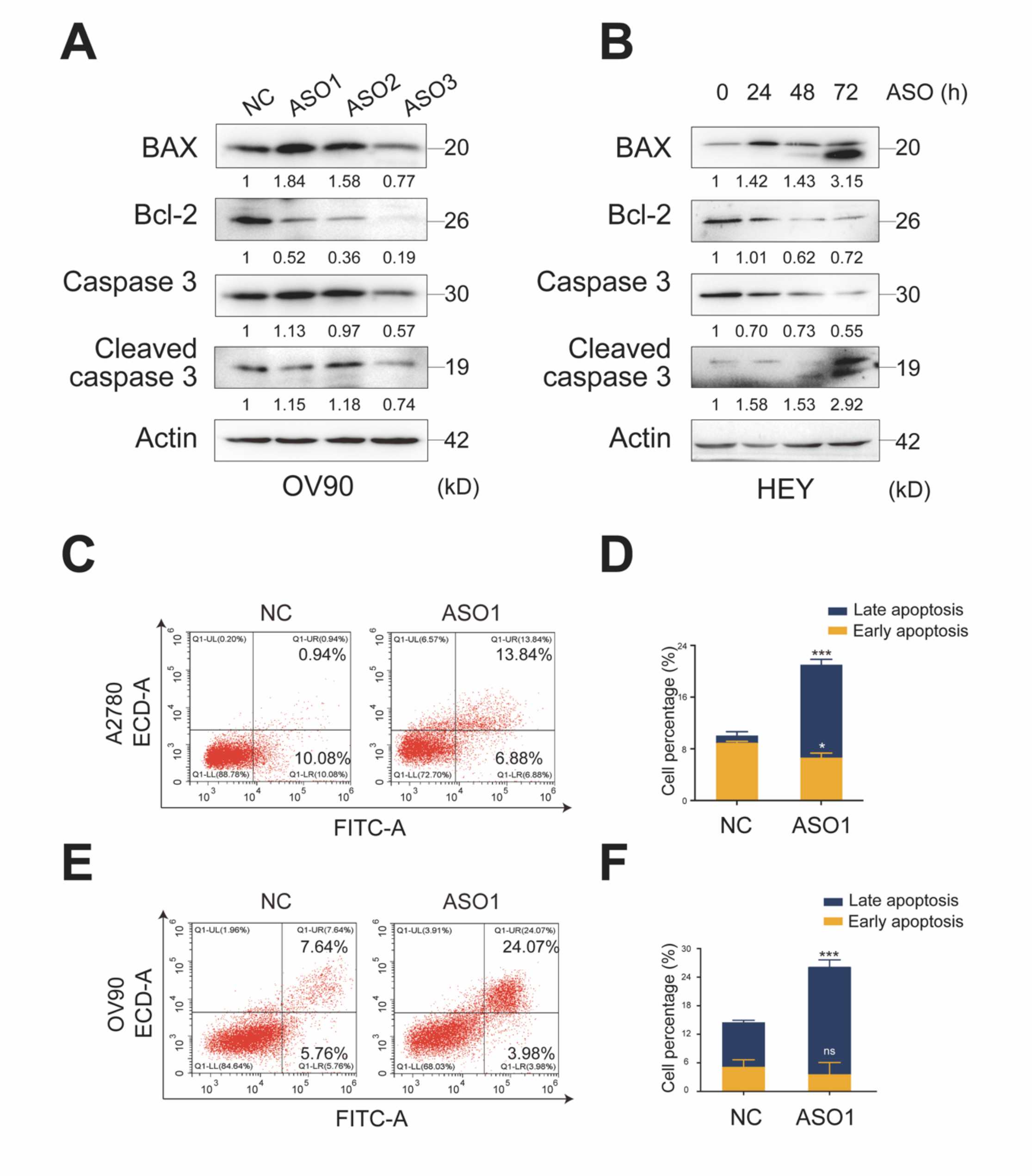

Fig. S7 Splice-switching antisense oligonucleotides can target BAX and regulate apoptosis in ovarian cancer cells, related to Fig. 7. A-B Western blot analysis of apoptosis markers in OV90 cells under different kinds of ASO treatment **(A)** and under ASO1 treatment with different time (0, 24, 48, and 72 h) **(B)**. **C-F** Apoptotic cells were detected by flow cytometry after staining with Annexin V/7-AAD in A2780 **(C, D)** and OV90 cells **(E, F)** treated with ASO1 (200 nM). P value was calculated by student's unpaired t-test. Three independent experiments were performed to gain mean ± SD value (n = 3 replicate experiments). **P* < 0.05, ***P* < 0.01, ****P* < 0.001.
