# Supplementary material for: Splicing Factor PQBP1 Curtails BAX Expression to Promote Ovarian Cancer Progression
Source: Adv Sci (Weinh). 2024 Feb 11;11(15):2306229. doi: 10.1002/advs.202306229 (PMC11022708; doi:10.1002/advs.202306229)
Supplement: Supplementary file 3 — Supplemental Table 2 [file ADVS-11-2306229-s005.pdf]

ADVANCED SCIENCE
Open Access

Supporting Information

for *Adv. Sci.*, DOI 10.1002/advs.202306229

Splicing Factor PQBP1 Curtails BAX Expression to Promote Ovarian Cancer Progression

Xihan Liu, Jiaojiao Zhang, Zixiang Wang, Mingyao Yan, Meining Xu, Gaoyuan Li, Victoria Shender, Jian-jun Wei, Jianqiao Li, Changshun Shao, Shiqian Zhang, Beihua Kong, Kun Song and Zhaojian Liu**

Table S2 Correlation of clinical characteristics with PQBP1 expression in ovarian cancer patients.

Clinical characteristics		PQBP1 expression			*P* value
		Total (n=149)	Low expression (n=42)	High expression (n=107)	
**Age (years)**	<56	74 (49.7)	18 (42.9)	56 (52.3)	0.298
	≥56	75 (50.3)	24 (57.1)	51 (47.7)	
**FIGO stage (2014)**	I and II	37 (24.8)	17 (40.5)	20 (18.7)	**0.006**
	III and IV	112 (75.2)	25 (59.5)	87 (81.3)	
**Histology**	HGSOC	140 (94.0)	35 (83.3)	105 (98.1)	**0.002**
	Non-HGSOC	9 (6.0)	7 (16.7)	2 (1.9)	
**CA-125 (U/mL)**	<760	70 (47.0)	24 (57.1)	46 (43.0)	0.129
	≥760	69 (46.3)	14 (33.3)	55 (51.4)	
	Unknown	10 (6.7)	4 (9.5)	6 (5.6)	
**Tumor diameter (cm)**	<8	50 (33.6)	18 (42.9)	32 (29.9)	0.287
	≥8	62 (41.6)	14 (33.3)	48 (44.9)	
	Unknown	37 (24.8)	10 (23.8)	27 (25.2)	
**Ascites involvement**	No	31 (20.8)	12 (28.6)	19 (17.8)	0.143
	Yes	118 (79.2)	30 (71.4)	88 (82.2)	
**Omental involvement**	No	53 (35.6)	23 (54.8)	30 (28.0)	**0.002**
	Yes	96 (64.4)	19 (45.2)	77 (72.0)	
**Residual disease (cm)**	<1	114 (76.5)	36 (85.7)	78 (72.9)	0.097
	≥1	35 (23.5)	6 (14.3)	29 (27.1)	
**Adjuvant chemotherapy**	No	20 (13.4)	9 (21.4)	11 (10.3)	0.072
	Yes	129 (86.6)	33 (78.6)	96 (89.7)	
**Platinum resistance relapse**	No	40 (26.8)	15 (35.7)	25 (23.4)	0.083
	Yes	20 (13.4)	2 (4.8)	18 (16.8)	
	Unknown	89 (59.7)	25 (59.5)	64 (59.8)	
**Death**	No	62 (41.6)	31 (73.8)	31 (29.0)	**<0.001**
	Yes	87 (58.4)	11 (26.2)	76 (71.0)	

Values are present as n (%) or median (range). FIGO, International Federation of Gynecology and Obstetrics; HGSOC, high-grade serous ovarian carcinoma; CA-125, Cancer Antigen 125.
