# Supplementary material for: Splicing Factor PQBP1 Curtails BAX Expression to Promote Ovarian Cancer Progression
Source: Adv Sci (Weinh). 2024 Feb 11;11(15):2306229. doi: 10.1002/advs.202306229 (PMC11022708; doi:10.1002/advs.202306229)
Supplement: Supplementary file 4 — Supplemental Table 3 [file ADVS-11-2306229-s001.pdf]

ADVANCED SCIENCE
Open Access

Supporting Information

for *Adv. Sci.*, DOI 10.1002/advs.202306229

Splicing Factor PQBP1 Curtails BAX Expression to Promote Ovarian Cancer Progression

Xihan Liu, Jiaojiao Zhang, Zixiang Wang, Mingyao Yan, Meining Xu, Gaoyuan Li, Victoria Shender, Jian-jun Wei, Jianqiao Li, Changshun Shao, Shiqian Zhang, Beihua Kong, Kun Song and Zhaojian Liu**

Table S3 Correlation between PQBP1 expression and clinicopathological characteristics

**Clinical characteristics**		**Univariate**		**Multivariate**	
		**HR (95%CI)**	***P***	**HR (95%CI)**	***P***
**PQBP1 expression**	Low	1		1	
	High	3.69	**<0.001**	3.49 (1.84-6.62)	**<0.001**
**Age (years)**	<56	1			
	≥56	0.97	0.870		
**FIGO stage (2014)**	I and II	1			
	III and IV	2.17	**0.006**		
**Histology**	HGSOC	1			
	Non-HGSOC	0.36	0.082		
**Grade**	II	1	0.111		
	III (poorly)	2.05	0.071		
	Unknown	3.15	0.051		
**CA-125 (U/mL)**	<760	1	0.575		
	≥760	1.19	0.428		
	Unknown	0.80	0.633		
**Tumor diameter (cm)**	<8	1	0.206		
	≥8	1.02	0.947		
	Unknown	1.53	0.118		
**Ascites involvement**	No	1			
	Yes	1.64	0.077		
**Omental involvement**	No	1			
	Yes	1.73	**0.020**		
**Residual disease (cm)**	<1	1			
	≥1	1.63	**0.036**		
**Adjuvant**	No	1			
	Yes	1.29	0.471		
**Platinum resistance**	No	1	**0.003**	1	**0.017**
	Yes	3.43	**0.001**	2.71 (1.32-5.56)	**0.006**
	Unknown	1.90	**0.024**	1.93 (1.10-3.37)	**0.021**

OS, overall survival; HR, hazard ratio; CI, confidence interval; FIGO, International Federation of Gynecology and Obstetrics; CA-125, Cancer Antigen 125.
